# Design, Synthesis, Characterization, and Molluscicidal Activity Screening of New Nicotinonitrile Derivatives against Land Snails, *M. cartusiana*

**DOI:** 10.3390/molecules27238284

**Published:** 2022-11-28

**Authors:** Hend M. A. Maaroof, Bander Albogami, Reham A. I. Abou-Elkhair, Abdalla E. A. Hassan, Fatma I. Al-Akhrasy, Salem A. A. El-Massry, Eman Fayad, Hamzah H. Ahmed, Islam Zaki

**Affiliations:** 1 Plant Protection Research Institute, Agricultural Research Center, Dokki, Giza 12513, Egypt; 2 Applied Nucleic Acids Research Center & Chemistry Department, Faculty of Science, Zagazig University, Zagazig 44523, Egypt; 3 Department of Biology, College of Sciences, Taif University, P.O. Box 11099, Taif 21944, Saudi Arabia; 4 Department of Biotechnology, College of Sciences, Taif University, P.O. Box 11099, Taif 21944, Saudi Arabia; 5 Radiologic Sciences Department, Faculty of Applied Medical Sciences, King Abdulaziz University, P.O. Box 80200, Jeddah 21589, Saudi Arabia; 6 Pharmaceutical Organic Chemistry Department, Faculty of Pharmacy, Port Said University, Port Said 42526, Egypt

**Keywords:** pyridine, nicotinonitrile, cyanopyridine, Mollusca, land snails, *M. cartusiana*, LC_50_, AChE, ALT, AST, TSP, histopathology

## Abstract

A new series of nicotinonitrile derivatives **2**–**7** was designed and synthesized from the starting material (*E*)-3-(4-chlorophenyl)-1-(4-methoxyphenyl)prop-2-en-1-one (**1**) to assess their molluscicidal activity. The newly synthesized nicotinonitrile compounds **2**–**7** were characterized based on FTIR, ^1^H-NMR, and ^13^C-APT NMR spectra as well as elemental microanalyses. The target compounds **2**–**7** were screened for their toxicity effect against *M. cartusiana* land snails and were compared to Acetamiprid as a reference compound. The results demonstrated that the nicotinonitrile-2-thiolate salts **4a** and **4b** had good mortality compared with that of Acetamiprid. The results of the in vivo effect of the prepared nicotinonitrile molecules **2**, **4a**, and **4b** on biochemical parameters, including AChE, ALT, AST, and TSP, indicated a reduction in the level of AChE and TSP as well as an increase in the concentration of transaminases (ALT and AST). A histopathological study of the digestive gland sections of the *M. cartusiana* land snails was carried out. The nicotinonitrile-2-thiolate salts **4a**,**b** showed vacuolization, causing the digestive gland to lose its function. It could be concluded that the water-soluble nicotinonitrile-2-thiolate salts **4a**,**b** could be adequate molluscicidal molecules against *M. cartusiana* land snails.

## 1. Introduction

Mollusca is the second largest phylum of the animal kingdom, and it is probably the third most important animal group after arthropods and vertebrates [1,2]. Land snails are considered one of the most destructive agricultural pests of numerous types of plants, including vegetables and horticultural crops [3,4]. They cause severe economic damage to a vast array of agricultural production, especially in areas with conditions suitable for their survival and dispersion [5,6]. Moreover, they can act as intermediate hosts for transmitting a lot of parasitic diseases infecting humans, animals, and birds [7,8]. Recently, in Egypt, a rapid increase in numerous species of land snails has been observed, infecting the majority of the vegetables and horticultural crops and causing severe damage to the field’s yield [9,10,11]. Several biological and chemical methods are being utilized for controlling land snails [12]. Chemical control is still one of the most effective strategies used to combat land snails [13]. However, the toxicity of these chemical molecules to non-target organisms and their environmental pollution limits their use [14]. Therefore, the search for new effective molluscicidal agents with safer environmental properties is increasingly required [15].

**Figure 1:**
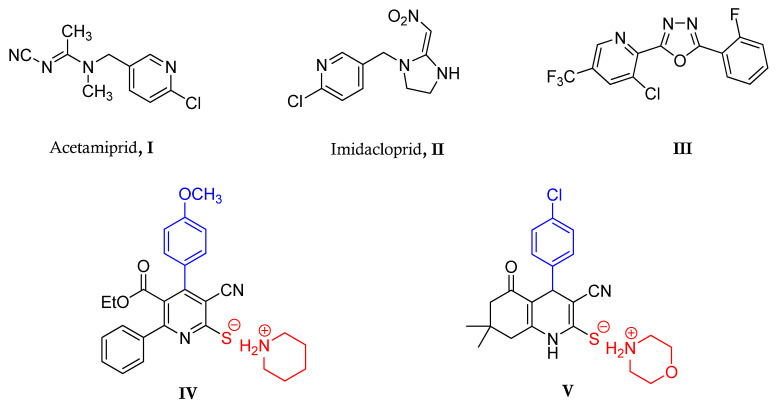
Pyridine **I**–**III** and nicotinonitrile **IV**, **V** derivatives as molluscicidal agents.

Pyridines broadly exist in nature and have various biological activities, including antimicrobial, anticancer, antioxidant, and insecticidal activities [16,17,18,19]. Nicotinonitrile (cyanopyridine) and its derivatives have been reported to possess tremendous biological activities, with insecticidal, antimicrobial, antiviral, and antioxidant properties [20,21,22,23]. Acetamiprid **I** and Imidacloprid **II** are pyridine-containing neonicotinoid derivatives with molluscicidal activity [24,25] (Figure 1). Acetamiprid **I** was shown to have molluscicidal activity against *Eobania vermiculata* (*E. vermiculata*) land snail species, while Imidacloprid **II** exhibited a toxic effect against the eggs of *E. vermiculata* [26,27]. Furthermore, trifluoromethyl pyridine molecule **III** had a potent toxic effect against *Mythimna separata* (*M. separata*), with LC_50_ of 42.7 mg/L, compared with that of Avermectin, with LC_50_ of 29.6 mg/L [28]. Interestingly, the bioassay results of piperidinium and morpholinium 3-cyanopyridine thiolate **IV** and **V** indicated strong aphidicidal activity against the adult cowpea aphid after a 24 h treatment, with LC_50_ values of 0.12 and 0.17 ppm, respectively, compared with that of Acetamiprid, with LC_50_ of 0.078 ppm [29].

**Figure 2:**
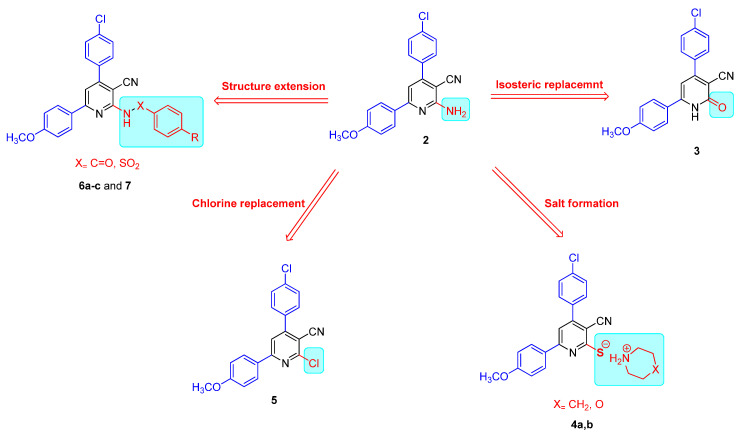
Design strategy of the prepared nicotinonitrile molecules **2**–**7**.

Given these observations, our study focused on the design and synthesis of nicotinonitrile derivatives in the hope that the new compounds would be beneficial as molluscicidal agents against *M. cartusiana* land snails. The basic structural feature of the lead template was formed from 2-aminonicotinonitrile **2**. Moreover, the 2-aminonicotinonitrile ring was decorated at C4 and C6 with 4-chlorophenyl and 4-methoxyphenyl rings. Furthermore, the amino group in the lead template **2** underwent isosteric replacement with either oxo (**3**) or chloro (**5**) atoms. In addition to that, the amino group was converted to the corresponding quaternary ammonium salt: piperidinium or morpholinium nicotinonitrile thiolate salts (**4a**,**b**). Further, the lead template **2** underwent further structural extension with either aryl amide (**6a**–**c**) or 4-methylbenzenesulphonamide (**7**) (Figure 2). In addition, the target nicotinonitrile molecules **2**–**7** were assessed for their toxic effects against *M. cartusiana* land snails. Additionally, some biochemical parameters of the most potent molecules were evaluated. Furthermore, a histopathological study of the digestive gland sections after treatment with the most potent molecules was also carried out.

## 2. Results and Discussion

### 2.1. Chemistry

**Scheme 1:**
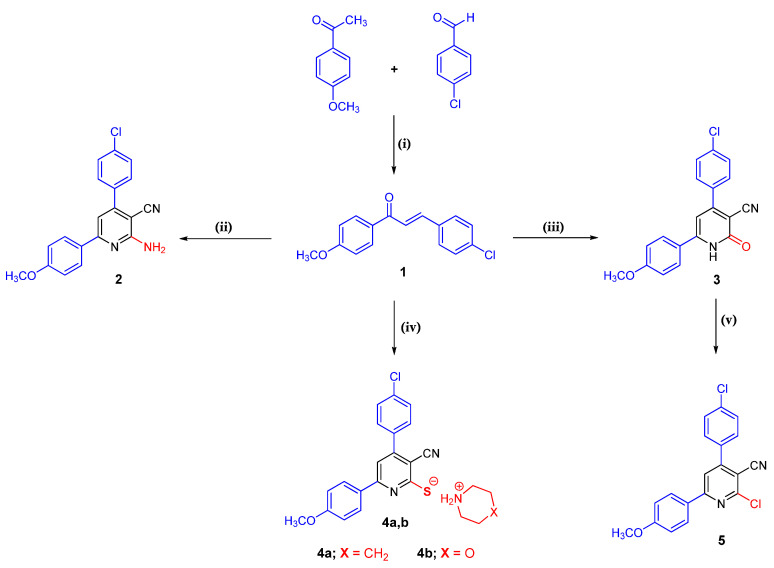
General synthetic route of nicotinonitrile compounds **1**–**5**. Reagents and reaction condition: (**i**) NaOH, EtOH, room temperature 12 h; (**ii**) CH_2_(CN)_2_, CH_3_COONH_4_, EtOH, reflux 12 h; (**iii**) CNCH_2_COOEt, CH_3_COONH_4_, EtOH, reflux 24 h; (**iv**) CNCH_2_CSNH_2_, respective base (piperidine or morpholine), EtOH, reflux overnight; (**v**) POCl_3_, *N,N*-dimethylaniline, reflux 3 days.

**Scheme 2:**
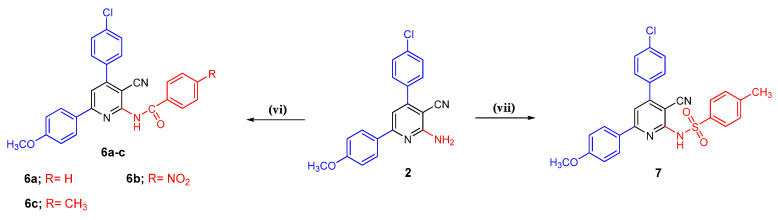
General synthetic route of nicotinonitrile compounds **6a**–**c** and **7**. Reagents and reaction condition: (**vi**) appropriate aroyl chloride, dry pyridine, heat at 70–80 °C for 3 days; (**vii**) 4-methylbenzenesulphonyl chloride, dry pyridine, heat at 70–80 °C for 3 days.

The strategy for synthesizing the target nicotinonitrile molecules is depicted in Scheme 1 and Scheme 2. Chalcone **1** was synthesized via the reaction of *p*-methoxy acetophenone and *p*-chlorobenzaldehyde in the presence of ethanolic NaOH [30]. 2-aminonicotinonitrile derivative **2** was synthesized by the cyclization of chalcone derivative **1** with malononitrile in refluxing pure ethanol in the presence of ammonium acetate. The structure of 2-aminonicotinonitrile **2** was confirmed on the basis of IR, ^1^H-NMR, and ^13^C-APT NMR spectra. The IR spectrum of compound **2** showed absorption bands at 3457, 3365, and 2205 cm^−1^, corresponding to the NH_2_ and CN groups, respectively. The ^1^H-NMR spectrum of compound **2** displayed three singlet peaks at *δ* 7.23, 6.97, and 3.82 ppm due to the protons of H-5 of pyridine ring, amino group, and OCH_3_, in addition to four doublet signals at *δ* 8.11, 7.70, 7.62, and 7.04 ppm, corresponding to the aromatic protons. The ^13^C-APT NMR spectrum of compound **2** showed peaks at *δ* 117.05, 114.01, and 85.49 ppm assigned for the nitrile group, C-5, and C-3 of the pyridine ring, respectively. 2-oxopyridine-3-carbonitrile derivative **3** was afforded by cyclization of the chalcone **1** with ethyl cyanoacetate in pure ethanol in the presence of ammonium acetate. The product was obtained as an amido tautomer, which is more stable than an imido tautomer. The structure of **3** was established by the presence of bands in the IR spectrum at 3502 and 1601 cm^−1^, characteristic of NH and C=O of the amide function, respectively. This was also confirmed by the presence of a singlet ^1^H-NMR peak at 12.70 ppm, corresponding to the amide NH (Scheme 1).

Nicotinonitrile-2-thiolate salts **4a**,**b** were obtained in good yields via the reaction of the chalcone **1** with 2-cyanothioacetamide in pure ethanol in the presence of an appropriate base, such as piperidine or morpholine, to give the thiolate salt derivatives **4a**,**b**, respectively. The ^1^H-NMR spectrum of compound **4b**, for example, revealed five singlet peaks at *δ* 7.85, 6.90, 3.85, 3.67, and 3.55 ppm due to the protons of H-5 pyridine ring, NH_2_^+^, OCH_3_, N^+^(CH_2_)_2_, and O(CH_2_)_2_ groups, respectively, in addition to four doublet signals at *δ* 8.23, 7.75, 7.67, and 7.09 ppm, attributable to the aromatic protons. The ^13^C-APT NMR spectrum of **4b** showed peaks at *δ* 115.82 and 100.80 ppm, corresponding to the nitrile group and C-3 of the pyridine ring, respectively, and two signals for four methylene carbon atoms at *δ* 66.21 and 49.08 ppm, corresponding to the methylene groups of the morpholine ring. In addition, the reaction of pyridine molecule **3** with POCl_3_ in the presence of *N*,*N*-dimethylaniline afforded the target 2-chloronicotinonitrile molecule **5**. The structure of the obtained compound **5** was confirmed on the basis of the spectral data. The ^1^H-NMR spectrum elicited the absence of the amidic NH proton signal; in addition, the ^13^C-APT NMR spectrum confirmed the carbon skeleton due to absence of signal, corresponding to C=O of the pyridone ring of the precursor molecule **3** (Scheme 1). Additionally, heating 2-aminonicotinonitrile **2** with the respective aroyl chloride derivatives in dry pyridine at 70–80 °C resulted in the formation of the corresponding nicotinonitril-2-yl aryl amide derivatives **6a**–**c**. The structure of amide molecules **6a**–**c** was substantiated on the basis of IR, ^1^H-NMR, and ^13^C-APT NMR spectra. The ^1^H-NMR spectrum of compound **6a**, for example showed the presence of a singlet peak at *δ* 8.36 ppm assigned for the NH group, as well as an additional signal at the aromatic region related to the phenyl group of the amide function. The ^13^C-APT NMR spectrum revealed a nitrile group at *δ* 114.70 ppm, while C-5 and C-3 of pyridine moiety appeared at *δ* 114.34 and 107.63 ppm, respectively. Under the same condition, 2-aminonicotinonitrile **2** reacted with 4-methylbenzenesulphonyl chloride to afford compound **7**. The structure of **6** was confirmed by spectral data (Scheme 2).

### 2.2. Biology

#### 2.2.1. Molluscicidal Activity

**Figure 3:**
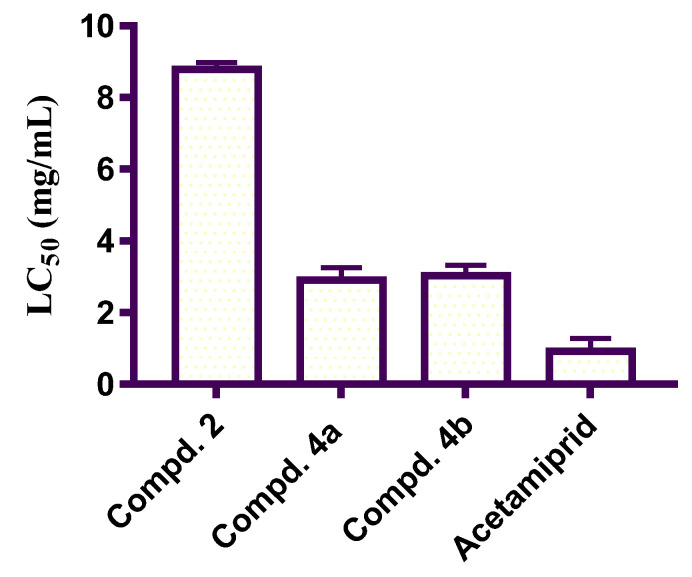
Calculated LC_50_ values (mg/mL) noted for active nicotinonitrile derivatives **2**, **4a**, **4b**, compared with that of Acetamiprid against *M. cartusiana* land snails.

The mortality of nicotinonitrile molecules **2**, **3**, **4a**–**b**, **5**, **6a**–**c** and **7** toward *M. cartusiana* land snails is screened and listed in Table 1. Acetamiprid was utilized as a reference control in our study. An insight inspection of the results revealed that piperidinium nicotinonitrile-2-thiolate **4a** and morpholinium nicotinonitrile-2-thiolate **4b** had a high mortality effect, 2-aminonicotinonitrile **2** had a moderate mortality effect, and 2-oxopyridine-3-carbonitrile **3**, nicotinonitril-2-yl aryl amides **6a**–**c**, and nicotinonitril-2-yl-4-methylbenzenesulphonamide **7** had no mortality activity. The LC_50_ values (lethal concentration for 50% of the population) for active nicotinonitrile molecules are calculated and listed in Table 1 and represented graphically in Figure 3. The nicotinonitrile-2-thiolate salts **4a**,**b** recorded LC_50_ values of 2.90 and 3.03 mg/mL, respectively, compared with that of Acetamiprid, with LC_50_ of 0.93 mg/mL. The results of toxicity screening experiments showed a correlation between the structural investigation of nicotinonitrile molecules **2**–**7** and their toxic effect against *M. cartusiana* land snails. In general, the nicotinonitrile molecule containing an amino substituent showed a better molluscicidal effect against *M. cartusiana* land snails than that of the compounds with other substituents. In addition, the replacement of the amino substituent with either aryl amide or the 4-methylbenzenesulphonamide group resulted in a complete loss of toxic effect against *M. cartusiana* land snails. Importantly, the corresponding quaternary ammonium salts, piperidinium or morpholinium nicotinonitrile-2-thiolate salts, exhibited a higher molluscicidal activity against *M. cartusiana* land snails than the rest of the compounds did. This may be attributed to presence of negative and positive charges, which increase the penetration through the cell membrane [31]. A comparison of the molluscicidal activity of the newly reported nicotinonitrile molecules revealed that nicotinonitrile-2-thiolate salts **4a**,**b** looked promising after further structural modification, which will be discussed in a future study.

#### 2.2.2. Biochemical Analysis

**Figure 4:**
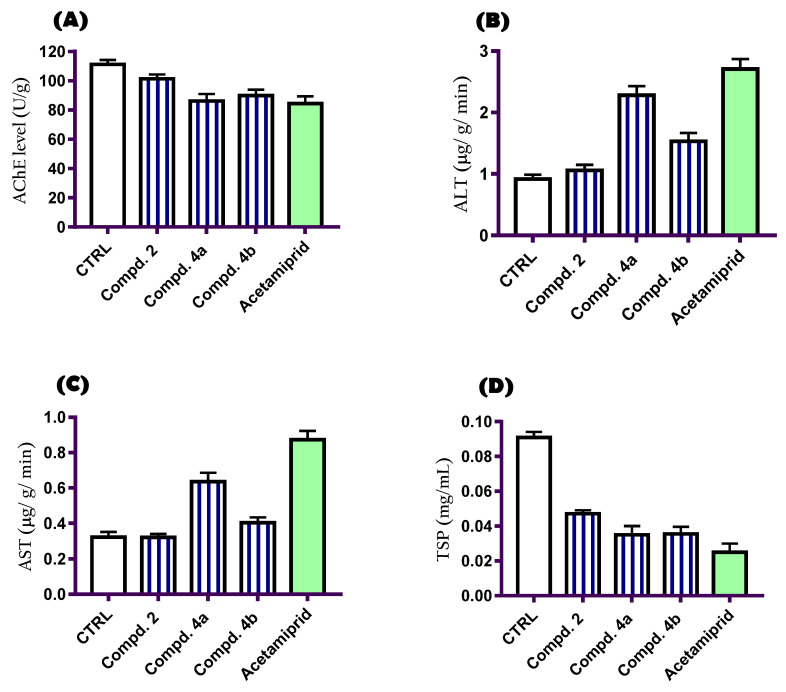
Impact of nicotinonitrile molecules **2, 4a**, **4b** and Acetamiprid on the levels of AChE, ALT, AST, and TSP at their LC_50_ values (mg/mL) in *M. cartusiana* land snails. (**A**) Effect of tested molecules on AChE; (**B**) effect of tested molecules on ALT; (**C**) effect of tested molecules on AST; (**D**) effect of tested molecules on TSP.

The effect of nicotinonitrile molecules **2**, **4a**, and **4b** on some biochemical parameters in *M. cartusiana* land snails was investigated. The levels of acetylcholinesterase (AChE), alanine transaminase (ALT), and aspartate aminotransferase (AST) activities as well as the total soluble protein (TSP) were measured following the treatment of *M. cartusiana* land snails with nicotinonitrile compounds **2**, **4a**, and **4b** at their LC_50_ values for 72 h. The results, demonstrated in Figure 4, elicited that compounds **2**, **4a**, and **4b** reduced the level of AChE by 1.09-, 1.29-, and 1.24-fold, respectively, compared with that of the no-treatment control (Figure 4A). Compounds **2** and **4b** reduced the AChE level, compared with Acetamiprid, while compound **4a** was found to be very close to that of Acetamiprid (1.31-fold less than the no-treatment control). On the contrary, compounds **2**, **4a**, and **4b** increased the level of ALT by 1.15-, 2.48-, and 1.66-fold, respectively, in comparison with that of the no-treatment control (Figure 4B). Likewise, the level of AST was increased, following the treatment with nicotinonitrile compounds **2**, **4a**, and **4b**, by 0.99-, 1.97-, and 1.25-fold, respectively, in comparison with that of the no-treatment control (Figure 4C). The increased levels of transaminases (ALT and AST) could be associated with the ability of the tested nicotinonitrile molecule to cause membrane destabilizing activity against *M. cartusiana* land snails. Moreover, nicotinonitrile compounds **2**, **4a**, and **4b** increased the level of TSP by 1.94-, 2.59-, and 2.56-fold, compared with that of the no-treatment control (Figure 4D).

#### 2.2.3. Histopathological Study

**Figure 5:**
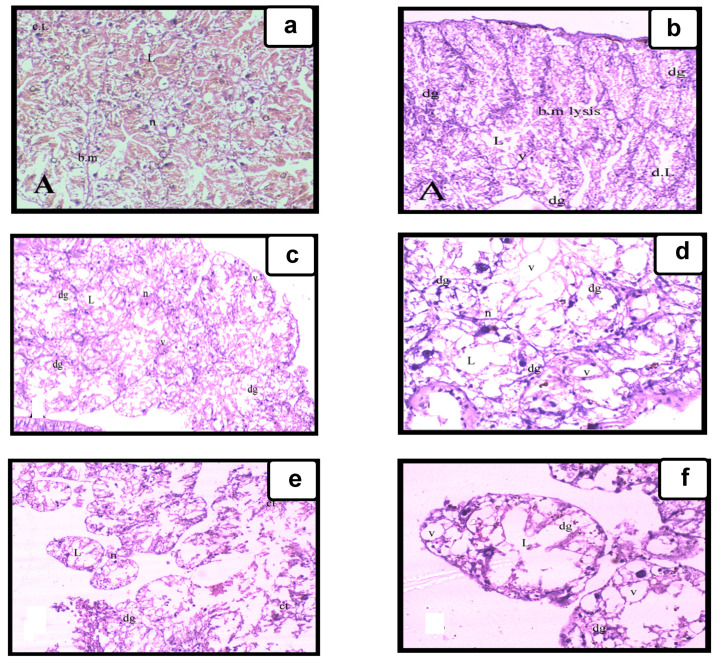
Histopathological examination of the digestive gland sections from land snail, *M. cartusiana*. (**a**) Photomicrograph of cross-section passing through the digestive gland of control land snail, *M. cartusiana*; (**b**) photomicrograph of cross-section passing through the digestive gland of land snail, *M. cartusiana*, that received 2-aminonicotinonitrile **2**; (**c**) photomicrograph of cross-section passing through the digestive gland of land snail, *M. cartusiana*, that received morpholinium nicotinonitrile-2-thiolate salt **4b** (×100); (**d**) photomicrograph of cross-section passing through the digestive gland of land snail, *M. cartusiana*, that received morpholinium nicotinonitrile-2-thiolate salt **4b** (×400); (**e**) photomicrograph of cross-section passing through the digestive gland of land snail, *M. cartusiana*, that received piperidinium nicotinonitrile-2-thiolate salt **4a** (×100); (**f**) photomicrograph of cross-section passing through the digestive gland of land snail, *M. cartusiana*, that received piperidinium nicotinonitrile-2-thiolate salt **4a** (×400). ct: connective tissue; bm: basement membrane; L: lumen; v: vacuole; n: nucleus; dg: digestive cell; bm lysis: basement membrane lysis; dL: dilated lumen cells.

The digestive gland of gastropods acts as the key organ for metabolism, as well as the accumulation and biotransformation of xenobiotics and chemicals [32]. The digestive gland of the control land snail, as given in Figure 5a, presented normal architecture with a bilobed tubule-acinar gland radiating from the dorsal portion on the back side of the stomach. It was surrounded by a thin membrane that consisted of a single layer of short columnar cells resting on a basal membrane with circular muscle fibers. However, the digestive gland sections of the tested land snail treated with 2-aminonicotinonitrile **2** had limited alterations, where the digestive envelope of the tubules appeared as in the untreated, tested land snails but a small elongation of the lumen occurred (Figure 5b). Additionally, more vacuoles formed in the tested land snail digestive gland received morpholinium nicotinonitrile-2-thiolate **4b** salt, and the binding connective tissue lost its shape and function. The lumen was dilated, the digestive cells suffered from degeneration, and the excretory cells ruptured, releasing the excretory granules out of the epithelial cell layer toward the lumen (Figure 5c,d). Moreover, the digestive gland sections of land snail given piperidinium nicotinonitrile-2-thiolate **4a** salt exhibited severe digestive gland damage, demonstrated in the form of losing the binding between the digestive gland acini, which are released individually. In addition, irreversible degeneration increased with wide, enormous vacuoles, causing the gland to lose its function. The vacuolization grew wide. The nucleus precipitated toward the basement membrane, and some erosion occurred after the treatment with piperidinium nicotinonitrile-2-thiolate salt **4a** (Figure 5e,f).

## 3. Conclusions

In this study, a new series of nicotinonitrile derivatives **2**–**7** were designed and synthesized from the starting material (*E*)-3-(4-chlorophenyl)-1-(4-methoxyphenyl)prop-2-en-1-one (**1**). The structure of these newly synthesized nicotinonitrile compounds **2**–**7** was characterized and confirmed using IR, ^1^H-NMR, and ^13^C-APT NMR spectra as well as elemental microanalyses. Bioassays were conducted for the molluscicidal activity of the synthesized nicotinonitrile compounds **2**–**7** against *M. cartusiana* land snails. The results revealed that nicotinonitrile-2-thiolate salts **4a** and **4b** had a good mortality effect (with LC_50_ values of 2.90 mg/mL and 3.03 mg/mL, respectively) compared with that of Acetamiprid (with LC_50_ of 0.93 mg/mL) as a reference compound. The results of the in vivo impact of nicotinonitrile molecules **2**, **4a**, and **4b** on biochemical biomarkers, including AChE, ALT, AST, and TSP, indicated a reduction in the levels of AChE and TSP as well as a significant rise in the concentrations of transaminases (ALT and AST). A histopathological study of the digestive gland sections of *M. cartusiana* land snails was carried out. Nicotinonitrile-2-thiolate salts **4a**,**b** showed vacuolization, causing the digestive gland to lose its function. It could be concluded that the water-soluble nicotinonitrile-2-thiolate salts **4a**,**b** could be adequate molluscicidal molecules against *M. cartusiana* land snails, which could be beneficial in the near future for further structural modification when searching for potent molluscicidal molecules.

## 4. Experimental

### 4.1. Chemistry

#### 4.1.1. General

To identify the melting points, ^1^H-NMR and ^13^C-APT NMR spectral data and elemental microanalyses were performed to substantiate the chemical structures of the prepared nicotinonitrile derivatives **2**–**7**. For experimental details, see Section S4.1.1 in the Supplementary Data.

#### 4.1.2. General Method for Synthesis of (*E*)-3-(4-Chlorophenyl)-1-(4-methoxyphenyl)prop-2-en-1-one (**1**)

To a well-stirred ethanolic solution of sodium hydroxide (0.0366 mol, 1.46 g), 4-methoxy acetophenone (0.0333 mol, 5 g) was added, and the mixture was stirred at room temperature for 10 min before an equimolar amount of 4-chlorobenzaldehyde (0.0333 mol, 4.68 g) was added. The reaction mixture was stirred at room temperature for 12 h. The solvent was evaporated under vacuum, and then the remained residue was suspended in water and neutralized with diluted. HCl. The obtained solid residue was filtered off, dried, and crystallized from methanol to give compound **1**.

White crystals (8.78 g, 96%), m.p. 102–104 °C. IR (ATR, cm^−1^): 3011 (νH aromatic), 2962 (νH aliphatic), and 1655 (νC=O). ^1^H-NMR (400 MHz, DMSO-*d*_6_, *δ* ppm): 8.01 (d, *J* = 8 Hz, 2H, arom. CH), 7.72 (d, *J* = 16 Hz, 1H, CH-olefinic), 7.54 (d, *J* = 8 Hz, 2H, arom. CH), 7.49 (d, *J* = 16 Hz, 1H, olefinic CH), 7.36 (d, *J* = 8 Hz, 2H, arom. CH), 6.96 (d, *J* = 8 Hz, 2H, arom. CH), and 3.87 (s, 3H, OCH_3_). ^13^C-APT NMR (100 MHz, DMSO-*d*_6_, *δ* ppm): 188.6 (CO), 163.8 (Ar), 142.6 (CH-olefinic), 136.4 (Ar), 133.8 (Ar), 131.0 (Ar), 131.0 (Ar), 129.7 (Ar), 129.4 (Ar), 122.5 (CH-olefinic), 114.1 (Ar), and 55.7 (OCH_3_). Anal. Calcd. for C_16_H_13_ClO_2_ (272.73): C, 70.46; H, 4.80. Found: C, 70.53; H, 4.84.

#### 4.1.3. General Method for Synthesis of 2-Amino-4-(4-chlorophenyl)-6-(4-methoxyphenyl)nicotinonitrile (**2**)

A mixture of compound **1** (0.011 mol, 3 g), malononitrile (0.011 mol, 720 mg), and excess ammonium acetate (0.22 mol, 17 g) was dissolved in absolute ethanol. The reaction mixture was heated under reflux temperature for 12 h. After cooling, the formed precipitate was filtered off, washed with ethanol, dried, and crystallized from absolute ethanol to get pure compound **2**.

Yellow crystals (2.71 g, 73%), m.p. 217–219 °C. IR (ATR, cm^−1^): 3457 (νN-H), 3365 (νN-H), and 2205 (νCN). ^1^H-NMR (400 MHz, DMSO-*d*_6_, *δ* ppm): 8.11 (d, *J* = 8.9 Hz, 2H, arom. CH), 7.70 (d, *J* = 8.6 Hz, 2H, arom. CH), 7.62 (d, *J* = 8.5 Hz, 2H, arom. CH), 7.23 (s, 1H, H-5 pyridine ring), 7.04 (d, *J* = 8.9 Hz, 2H, arom. CH), 6.97 (s, 2H, NH_2_), and 3.82 (s, 3H, OCH_3_). ^13^C-APT NMR (100 MHz, DMSO-*d*_6_, *δ* ppm): 161.1 (C-2 pyridine ring), 160.8 (Ar), 158.4 (Ar), 153.4 (Ar), 135.9 (Ar), 134.4 (Ar), 130.3 (Ar), 129.8 (Ar), 128.9 (Ar), 128.7 (Ar), 117.1 (C≡N), 114.0 (C-5 pyridine ring), 108.3 (Ar), 85.5 (C-3 pyridine ring), and 55.3 (OCH_3_). Anal. Calcd. for C_19_H_14_ClN_3_O (335.79): C, 67.96; H, 4.20; N, 12.51. Found: C, 68.06; H, 4.24; N, 12.42.

#### 4.1.4. General Method for Synthesis of 4-(4-Chlorophenyl)-6-(4-methoxyphenyl)-2-oxo-1,2-dihydropyridine-3-carbonitrile (**3**)

A mixture of compound **1** (1.83 mmol, 0.50 g), ethyl cyanoacetate (2.75 mmol, 300 L), and 2.8 g of ammonium acetate in absolute ethanol (20 mL) was heated under reflux for 24 h. After completion of the reaction, the excess solvent was concentrated under vacuum, and the formed product was filtered off, dried, and crystallized from absolute ethanol to afford pure compound **3**.

Yellow solid (0.46 g, 65%), m.p. 194–196 °C. IR (ATR, cm^−1^): 3493 (νN-H), 2218 (νCN), and 1740 (νC=O, amide). ^1^H-NMR (400 MHz, DMSO-*d*_6_, *δ* ppm): 12.70 (s, 1H, NH), 7.90 (d, *J* = 8.7 Hz, 2H, arom. CH), 7.75 (d, *J* = 8.5 Hz, 2H, arom. CH), 7.64 (d, *J* = 8.5 Hz, 2H, arom. CH), 7.07 (d, *J* = 8.9 Hz, 2H, arom. CH), 6.79 (s, 1H, H-5 pyridone ring), and 3.84 (s, 3H, OCH_3_). ^13^C-APT NMR (100 MHz, DMSO-*d*_6_, *δ* ppm): 161.8 (C=O), 135.2 (Ar), 135.0 (Ar), 130.2 (Ar), 129.5 (Ar), 128.8 (Ar), 116.5 (C≡N), 114.4 (C-5 pyridone ring), and 55.5 (OCH_3_). Anal. Calcd. for C_19_H_13_ClN_2_O_2_ (336.77): C, 67.76; H, 3.89; N, 8.32. Found: C, 67.81; H, 3.84; N, 8.19.

#### 4.1.5. General Method for Synthesis of 4-(4-Chlorophenyl)-3-cyano-6-(4-methoxyphenyl)pyridine-2-thiolate salts **4a**,**b**

To a solution of compound **1** (0.366 mmol, 100 mg) and 2-cyanothioactamide (0.549 mmol, 54.9 mg) in pure ethanol, the respective base (0.549 mmol) was added. The reaction mixture was heated to reflux temperature overnight. After completion of the reaction, the reaction mixture was poured onto a beaker containing ice water. A solid formed, and it was filtered off and crystallized from pure ethanol to give pure compound **4a**,**b**.

##### Piperidinium 4-(4-Chlorophenyl)-3-cyano-6-(4-methoxyphenyl)pyridine-2-thiolate (**4a**)

White crystal (70 mg, 43%), m.p. 255–257 °C. IR (ATR, cm^−1^): 3453 (νNH), 3291 (νNH), and 2163 (νCN). ^1^H-NMR (400 MHz, DMSO-*d*_6_, *δ* ppm): 8.26 (d, *J* = 8.7 Hz, 2H, arom. CH), 7.85 (s, 1H, H-5 pyridine ring), 7.75 (d, *J* = 8.4 Hz, 2H, arom. CH), 7.67 (d, *J* = 8.4 Hz, 2H, arom. CH), 7.07 (d, *J* = 8.7 Hz, 2H, arom. CH), 6.74 (s, 2H, NH_2_^+^), 3.85 (s, 3H, OCH_3_), 3.52 (s, 4H, CH_2_N^+^CH_2_), and 1.60 (s, 6H, 3CH_2_). ^13^C-APT NMR (100 MHz, DMSO-*d*_6_, *δ* ppm): 167.1 (C-2 pyridine ring), 164.9 (Ar), 161.6 (Ar), 157.9 (Ar), 153.0 (s), 135.0 (Ar), 134.8 (Ar), 130.6 (Ar), 129.3 (Ar), 129.3 (Ar), 128.9 (Ar), 124.4 (Ar), 115.8 (C≡N), 115.6 (Ar), 114.3 (C-5 pyridine ring), 100.7 (C-3 pyridine ring), 55.4 (OCH_3_), 49.7 (CH_2_N^+^CH_2_), 25.8 (CH_2_CH_2_ N^+^), and 24.0- (CH_2_CH_2_CH_2_). Anal. Calcd. for C_24_H_24_ClN_3_OS (437.98): C, 65.81; H, 5.52; N, 9.59. Found: C, 65.89; H, 5.59; N, 9.47.

##### Morpholinium 4-(4-Chlorophenyl)-3-cyano-6-(4-methoxyphenyl)pyridine-2-thiolate (**4b**)

White crystal (79 mg, 49%), m.p. 262–264 °C. IR (ATR, cm^−1^): 3301 (νNH), 3205 (νNH), and 2156 (νCN). ^1^H-NMR (400 MHz, DMSO-*d*_6_, *δ* ppm): *δ* 8.23 (d, *J* = 8.6 Hz, 2H, arom. CH), 7.85 (s, 1H, H-5 pyridine ring), 7.75 (d, *J* = 8.4 Hz, 2H, arom. CH), 7.67 (d, *J* = 8.4 Hz, 2H, arom. CH), 7.09 (d, *J* = 8.7 Hz, 2H, arom. CH), 6.90 (s, 2H, NH_2_^+^), 3.85 (s, 3H, OCH_3_), 3.67 (s, 4H, CH_2_OCH_2_), and 3.55 (s, 4H, CH_2_N^+^CH_2_). ^13^C-APT NMR (100 MHz, DMSO-*d*_6_, *δ* ppm): 167.4 (C-2 pyridine ring), 164.5 (Ar), 161.6 (Ar), 158.0- (Ar), 153.0- (Ar), 135.0 (Ar), 134.8 (Ar), 130.6 (Ar), 129.3 (Ar), 129.0 (Ar), 124.2 (Ar), 115.8 (C≡N), 115.7 (Ar), 114.4 (Ar), 100.8- (C-3 pyridine ring), 66.2- (CH_2_OCH_2_), 55.5- (OCH_3_), and 49.1 (CH_2_N^+^CH_2_). Anal. Calcd. for C_23_H_22_ClN_3_O_2_S (439.96): C, 62.79; H, 5.04; N, 9.55. Found: C, 62.68; H, 4.97; N, 9.63.

#### 4.1.6. General Method for Synthesis of 2-Chloro-4-(4-chlorophenyl)-6-(4-methoxyphenyl)nicotinonitrile (**5**)

Compound **3** (0.595 mmol, 200 mg) was dissolved in 5 mL of phosphorous oxychloride, and 0.5 mL of *N*,*N* dimethylaniline was added by drops. The reaction mixture was refluxed for 3 days. The reaction mixture was poured onto ice water. The obtained solid residue was filtered off, dried, and crystallized from absolute ethanol to give pure compound **5**.

White powder (160 mg, 74%), m.p. 157–159 °C. IR (ATR, cm^−1^): 3076 (νH aromatic), 2938 (νH aliphatic), and 2224 (νCN). ^1^H-NMR (400 MHz, DMSO-*d*_6_, *δ* ppm): 8.23 (d, *J* = 8.9 Hz, 2H, arom. CH), 8.19 (s, 1H, H-5 pyridine ring), 7.82 (d, *J* = 8.5 Hz, 2H, arom. CH), 7.70 (d, *J* = 8.5 Hz, 2H, arom. CH), 7.11 (d, *J* = 9.0 Hz, 2H, arom. CH), and 3.86 (s, 3H, OCH_3_). ^13^C-APT NMR (100 MHz, DMSO-*d*_6_, *δ* ppm): 162.0 (C-2 pyridine ring), 154.9 (Ar), 134.1 (Ar), 130.8 (Ar), 129.5 (Ar), 128.9 (Ar), 127.8 (Ar), 118.5 (Ar), 115.3 (C≡N), 114.6 (C-5 pyridine ring), 105.3 (C-3 pyridine ring), and 55.5 (OCH_3_). Anal. Calcd. for C_19_H_12_Cl_2_N_2_O (355.22): C, 64.24; H, 3.41; N, 7.89. Found: C, 64.34; H, 3.49; N, 7.78.

#### 4.1.7. General Method for Synthesis of *N*-(4-(4-Chlorophenyl)-3-cyano-6-(4-methoxyphenyl)pyridin-2-yl)aryl amides **6a**–**c**

A mixture of compound **2** (0.44 mmol, 150 mg) and appropriate aroyl chloride (1.322 mmol) was dissolved in dry pyridine. The reaction mixture was heated at 60–70 °C for 3 days. After the completion of the reaction, the whole reaction mixture was poured onto ice water with continuous stirring, and the water phase was extracted with ethyl acetate. The combined organic extracts were washed with water and dried with Na_2_SO_4_, and the solvent was removed under reduced pressure. The precipitated solid product was filtered off and purified by either crystallization or column chromatography.

##### *N*-(4-(4-Chlorophenyl)-3-cyano-6-(4-methoxyphenyl)pyridin-2-yl)benzamide (**6a**)

Yellow crystals (143 mg, 74%), crystallized from absolute ethanol, m.p. 211–213 °C. IR (ATR, cm^−1^): 3261 (νN-H), 2218 (νCN) and 1668 (νC=O, amide). ^1^H-NMR (400 MHz, DMSO-*d*_6_, *δ* ppm): 11.33 (s, 1H, NH), 8.25 (d, *J* = 8.9 Hz, 2H, arom. CH), 8.09–8.06 (m, 3H, arom. CH and H-5 pyridine ring), 7.81 (d, *J* = 8.6 Hz, 2H, arom. CH), 7.70 (d, *J* = 8.6 Hz, 2H, arom. CH), 7.60–7.57 (m, 2H, arom. CH), 7.10 (d, *J* = 9.0 Hz, 2H, arom. CH), and 3.85 (s, 3H, OCH_3_). ^13^C-APT NMR (100 MHz, DMSO-*d*_6_, *δ* ppm): 166.1 (CO), 161.6 (C-2 pyridine ring), 158.4 (Ar), 153.9 (Ar), 153.5 (Ar), 135.0 (Ar), 134.8 (Ar), 132.9 (Ar), 132.5 (Ar), 130.7 (Ar), 129.3 (Ar), 129.2 (Ar), 129.0 (Ar), 128.8 (Ar), 128.6 (Ar), 128.2 (Ar), 116.8 (Ar), 115.8 (C≡N), 114.3 (C-5 pyridine ring), 100.6 (C-3 pyridine ring), and 55.4 (OCH_3_). Anal. Calcd. for C_26_H_18_ClN_3_O_2_ (439.89): C, 70.99; H, 4.12; N, 9.55. Found: C, 71.08; H, 4.06; N, 9.47.

##### *N*-(4-(4-Chlorophenyl)-3-cyano-6-(4-methoxyphenyl)pyridin-2-yl)-4-nitrobenzamide (**6b**)

White crystals (150 mg, 70%), crystallized from ethyl acetate, m.p. 223–225 °C. IR (ATR, cm^−1^): 3222 (νN-H), 2222 (νCN), and 1666 (νC=O, amide). ^1^H-NMR (400 MHz, DMSO-*d*_6_, *δ* ppm): 11.71 (s, 1H, NH), 8.42 (d, *J* = 8.9 Hz, 2H, arom. CH), 8.27 (dd, *J* = 15.1, 8.9 Hz, 4H, arom. CH), 8.10 (s, 1H, H-5 pyridine ring), 7.82 (d, *J* = 8.6 Hz, 2H, arom. CH), 7.71 (d, *J* = 8.6 Hz, 2H, arom. CH), 7.10 (d, *J* = 9.0 Hz, 2H, arom. CH), and 3.85 (s, 3H, OCH_3_). ^13^C-APT NMR (100 MHz, DMSO-*d*_6_, *δ* ppm): 164.8 (CO), 161.6 (C-2 pyridine ring), 158.5 (Ar), 153.6 (Ar), 153.4 (Ar), 149.7 (Ar), 138.6 (Ar), 135.1 (Ar), 134.7 (Ar), 130.8 (Ar), 129.7 (Ar), 129.3 (Ar), 129.0 (Ar), 128.7 (Ar), 123.8 (Ar), 117.1- (Ar), 115.7 (C≡N), 114.4 (C-5 pyridine ring), 101.5 (C-3 pyridine ring), and 55.4- (OCH_3_). Anal. Calcd. for C_26_H_17_ClN_4_O_4_ (484.89): C, 64.40; H, 3.53; N, 11.55. Found: C, 64.32; H, 3.61; N, 11.59.

##### *N*-(4-(4-Chlorophenyl)-3-cyano-6-(4-methoxyphenyl)pyridin-2-yl)-4-methylbenzamide (**6c**)

White powder (160 mg, 80%), the residue was purified with column chromatography (methylene chloride/hexane), m.p. 207–209 °C. IR (ATR, cm^−1^): 3561 (νN-H), 2218 (νCN) and 1697 (νC=O, amide); ^1^H-NMR (400 MHz, DMSO-*d*_6_, *δ* ppm): 8.11 (s, 1H, NH), 7.93 (d, *J* = 9.0 Hz, 2H, arom. CH), 7.83 (d, *J* = 8.6 Hz, 2H, arom. CH), 7.75 (d, *J* = 8.2 Hz, 3H, arom. CH and H-5 pyridine), 7.71 (d, *J* = 8.6 Hz, 2H, arom. CH), 7.32 (d, *J* = 8.0 Hz, 2H, arom. CH), 7.01 (d, *J* = 9.0 Hz, 2H, arom. CH), 3.80 (s, 3H, OCH_3_), and 2.33 (s, 3H, CH_3_). ^13^C-APT NMR (100 MHz, DMSO-*d_6_*, *δ* ppm): 172.5- (CO), 161.8- (C-2 pyridine ring), 158.2- (Ar), 156.0- (Ar), 154.0- (Ar), 143.9- (Ar), 135.5 (Ar), 134.0- (Ar), 131.0 (Ar), 130.8 (Ar), 129.7- (Ar), 129.2 (Ar), 129.1 (Ar), 128.9 (Ar), 127.8 (Ar), 118.0 (Ar), 115.3 (C≡N), 114.4 (C-5 pyridine ring), 101.6- (C-3 pyridine ring), 55.4- (OCH_3_), and 21.1 (CH_3_). Anal. Calcd. for C_27_H_20_ClN_3_O_2_ (453.92): C, 71.44; H, 4.44; N, 9.26. Found: C, 71.35; H, 4.52; N, 9.33.

#### 4.1.8. General Method for Synthesis of *N*-(4-(4-Chlorophenyl)-3-cyano-6-(4-methoxyphenyl)pyridin-2-yl)-4-methylbenzenesulfonamide (**7**)

A mixture of compound **2** (0.44 mmol, 150 mg) and 4-methylbenzenesulphonyl chloride (1.322 mmol, 252 mg) was dissolved in dry pyridine. The reaction mixture was heated at 60–70 °C for 3 days. After the completion of the reaction, the whole reaction mixture was poured onto ice water with continuous stirring, and the water phase was extracted with ethyl acetate. The combined organic extracts were washed with water and dried with Na_2_SO_4_, and the solvent was removed under reduced pressure. The obtained residue was purified with column chromatography (methylene chloride/hexane) to give pure compound **7**.

White powder (160 mg, 74%), m.p. 228–230 °C. IR (ATR, cm^−1^): 3426 (νN-H), 2224 (νCN); ^1^H-NMR (400 MHz, DMSO-*d*_6_, *δ* ppm): 8.36 (s, 1H, NH), 7.86–7.81 (m, 3H, arom. CH and H-5 pyridine ring), 7.78–7.67 (m, 5H, arom. CH), 7.42 (d, *J* = 8.3 Hz, 3H, arom. CH), 7.04 (d, *J* = 8.9 Hz, 2H, arom. CH), 3.87 (s, 3H, OCH_3_), and 3.32 (s, 3H, CH_3_). ^13^C-APT NMR (100 MHz, DMSO-*d*_6_, *δ* ppm): *δ* 162.0 (C-2 pyridine ring), 158.3 (Ar), 154.5 (Ar), 149.9 (Ar), 146.1 (Ar), 135.5 (Ar), 134.5 (Ar), 133.8 (Ar), 130.9 (Ar), 129.0 (Ar), 129.4 (Ar), 129.1 (Ar), 129.0 (Ar), 127.5 (Ar), 120.6 (Ar), 114.7 (C≡N), 114.3 (C-5 pyridine ring), 107.6 (C-3 pyridine ring), 55.5 (OCH_3_), and 21.2 (CH_3_). Anal. Calcd. for C_26_H_20_ClN_3_O_3_S (489.97): C, 63.73; H, 4.11; N, 8.58. Found: C, 63.82; H, 4.16; N, 8.50.

### 4.2. Biological Studies

#### 4.2.1. Molluscicidal Activity against *M. cartusiana* Land Snails

The molluscicidal activity test was conducted according to the guidelines of the World Health Organization (WHO), with a slight modification through the use of two mixed solvents to dissolve the nicotinonitrile compounds in order to study the effect of nicotinonitrile molecules **2**–**7** on *M. cartusiana* land snails [33]. *Monacha cartusiana* snails were collected from the field of Sobah village, Hehia district, Sharkia governorate, maintained under laboratory conditions for two weeks prior to the tests, and fed daily with lettuce leaves for acclimatization. A series of concentrations, that is, four concentrations of each compound (1, 2, 3, and 4 mg/mL), were prepared by mixing an appropriate amount of each compound with one drop of Tween 80 and one drop of DMSO until the compounds became completely soluble, followed by the addition of the appropriate volume of water to provide homogeneous suspension. Before starting the experiment, the snails were starved for 48 h. The snails were irradiated with water for activation. Thirty snails for each concentration were immersed in the corresponding solution of the tested compound for 30 s. A piece of fresh lettuce leaf was dipped for 30 s, and then every ten snails were introduced in a box supplied with a disc of lettuce. The boxes were covered with a muslin cloth and secured with a rubber band to prevent the snails’ escape. Ten snails were utilized as a control by immersing them in water containing one drop of Tween 80 and one drop of DMSO. A lack of contraction indicated death, and the dead snails were removed. The mortality percentage was calculated after 3 days and corrected by Abbott’s Formula. The LC_50_ values (lethal concentration for 50% of the population) for the active compounds were calculated.

#### 4.2.2. Biochemical Assays

The in vivo impact of nicotinonitrile compounds **2**, **4a**, **4b** and Acetamiprid on the levels of AChE, ALT, AST as well as the TSP activities in *M. cartusiana* snails was determined [34,35,36]. See Section S4.2.2 in the Supplementary Data.

#### 4.2.3. Histological Study

A histopathological study of the digestive gland sections of the *M. cartusiana* land snails was conducted. They were observed by light microscopy after treatment with nicotinonitrile compounds **2**, **4a**, and **4b** compared with that of the no-treatment control [37,38]. After treating land snails with nicotinonitrile molecules **2**, **4a**, and **4b** with a concentration equal to the LC_50_ values, histological studies were performed. The shell of each snail was carefully broken, and the soft tissues were dissected out. The digestive gland and the intestines of the snail were placed in a petri dish containing isotonic buffer. The digestive gland was separated and fixed in 10% formalin. For histological studies, the organ was dehydrated using an ascending series of ethanol alcohol, cleared in xylene for 2 min, and then immersed in three changes: The first consisted of xylene + wax in ratio 1:1, and the second and the third are wax each for 1/2 h. Embedding in paraffin and blocking was carried out under vacuum. Serial transverse sections of 6–8 μm were mounted on clean slides without using any adhesive material. Ehleish’s haematoxylin and eosin were employed for general histological studies of the digestive gland.

## Figures and Tables

**Table 1:** Mortality and calculated LC_50_ values (mg/mL) noted for active nicotinonitrile derivatives **2**–**7** and Acetamiprid against *M. cartusiana* land snails.

Compd. No.	Conc. (mg/mL)	Mortality	LC_50_ (mg/mL)
**2**	1	1	8.78 ± 0.86
	2	2	
	3	3	
	4	3	
**3**	1	0	NA
	2	0	
	3	0	
	4	0	
**4a**	1	2	2.90 ± 0.25
	2	4	
	3	4	
	4	6	
**4b**	1	2	3.03 ± 0.27
	2	3	
	3	4	
	4	7	
**5**	1	0	NA
	2	1	
	3	1	
	4	2	
**6a**	1	0	NA
	2	0	
	3	0	
	4	0	
**6b**	1	0	NA
	2	0	
	3	0	
	4	0	
**6c**	1	0	NA
	2	0	
	3	0	
	4	0	
**7**	1	0	NA
	2	0	
	3	0	
	4	0	
**Acetamiprid**	1	5	0.93 ± 0.11
	2	6	
	3	6	
	4	7	

NA: not active.

## Data Availability

Data is contained within the article and Supplementary Data.

## Supplementary Materials

The following supporting information can be downloaded at: https://www.mdpi.com/article/10.3390/molecules27238284/s1, Figure S1: FTIR spectrum of compound **1**, Figure S2: ^1^H-NMR spectrum of compound **1**, Figure S3: ^13^C-NMR spectrum of compound **1,** Figure S4: FTIR spectrum of compound **2**, Figure S5: ^1^H-NMR spectrum of compound **2**, Figure S6: ^13^C-NMR spectrum of compound **2,** Figure S7: FTIR spectrum of compound **3**, Figure S8: ^1^H-NMR spectrum of compound **3**, Figure S9: ^13^C-NMR spectrum of compound **3,** Figure S10: FTIR spectrum of compound **4**a, Figure S11: ^1^H-NMR spectrum of compound **4**a, Figure S12: ^13^C-NMR spectrum of compound **4a,** Figure S13: FTIR spectrum of compound **4b**, Figure S14: ^1^H-NMR spectrum of compound **4b**, Figure S15: ^13^C-NMR spectrum of compound **4b,** Figure S16: FTIR spectrum of compound **5**, Figure S17: ^1^H-NMR spectrum of compound **5**, Figure S18: ^13^C-NMR spectrum of compound **5,** Figure S19: FTIR spectrum of compound **6a**, Figure S20: ^1^H-NMR spectrum of compound **6a**, Figure S21: ^13^C-NMR spectrum of compound **6a,** Figure S22: FTIR spectrum of compound **6b**, Figure S23: ^1^H-NMR spectrum of compound **6b**, Figure S24: ^13^C-NMR spectrum of compound **6b,** Figure S25: FTIR spectrum of compound **6c**, Figure S26: ^1^H-NMR spectrum of compound **6c**, Figure S27: ^13^C-NMR spectrum of compound **6c,** Figure S28: FTIR spectrum of compound **7**, Figure S29: ^1^H-NMR spectrum of compound **7**, Figure S30: ^13^C-NMR spectrum of compound **7.**

## Sample Availability

Samples of the compounds are available from the authors.
