# Supplementary material for: Design, Synthesis, Characterization, and Molluscicidal Activity Screening of New Nicotinonitrile Derivatives against Land Snails, M. cartusiana
Source: Molecules. 2022 Nov 28;27(23):8284. doi: 10.3390/molecules27238284 (PMC9740234; doi:10.3390/molecules27238284)
Supplement: Supplementary file 1 [file molecules-27-08284-s001.zip › molecules-1998651-supplementary.pdf]

Supporting Information

Design, Synthesis, Characterization, and Molluscicidal Activity Screening of New Nicotinonitrile derivatives against Land Snails*, M. cartusiana*

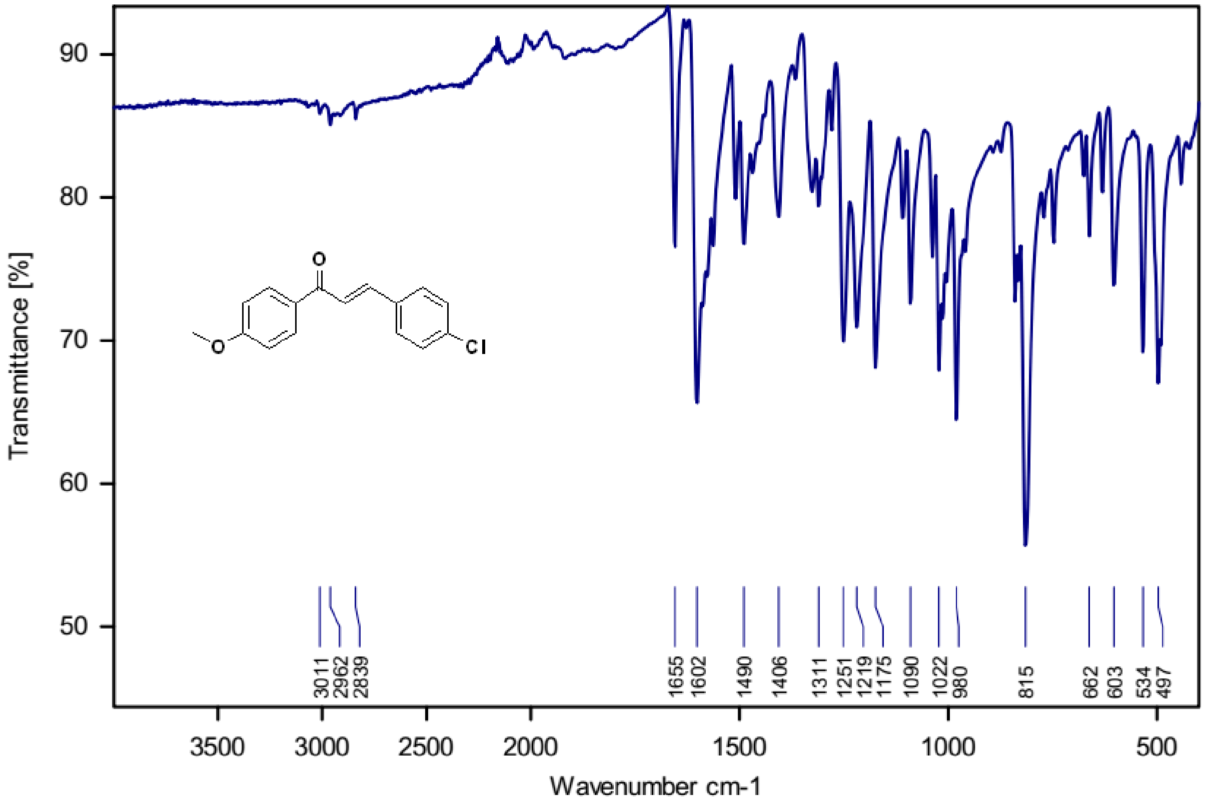


**Figure S1:** FTIR spectrum of compound **1**.

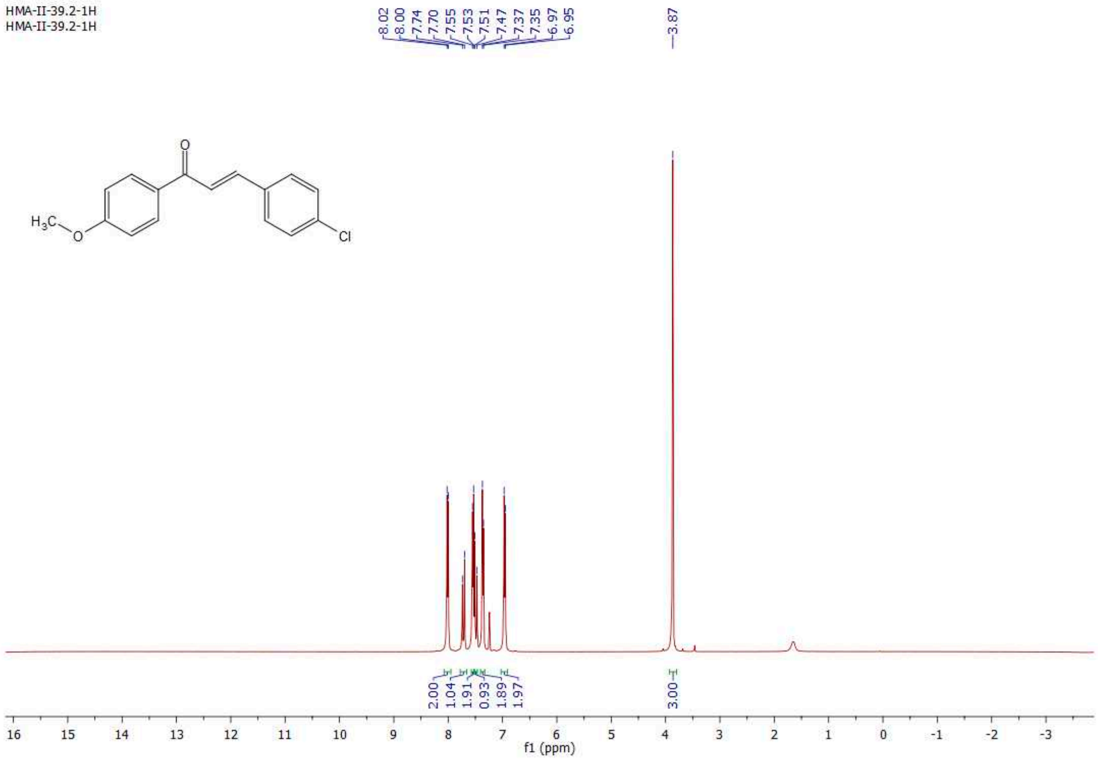


**Figure S2:** $^{1}$H-NMR spectrum of compound **1**.

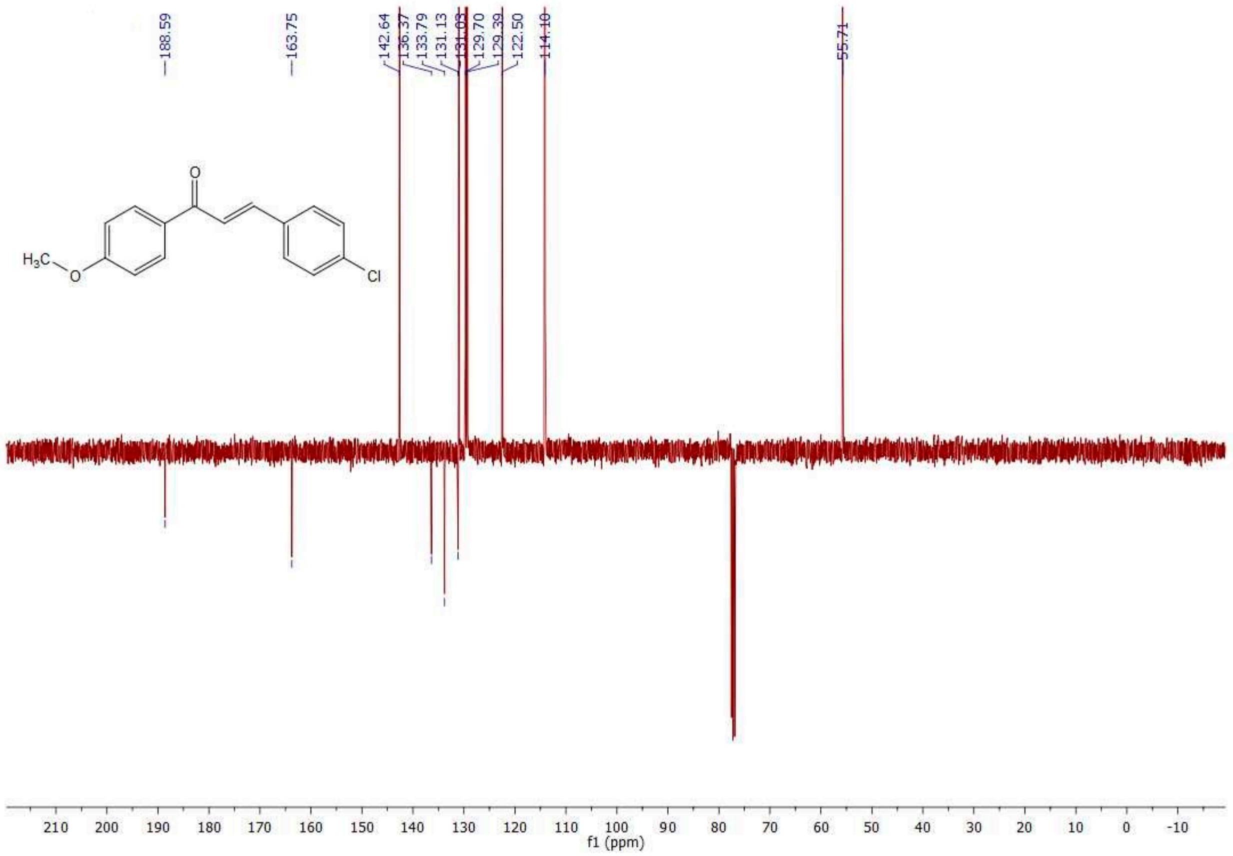


**Figure S3:** $^{13}C$-NMR spectrum of compound **1**.

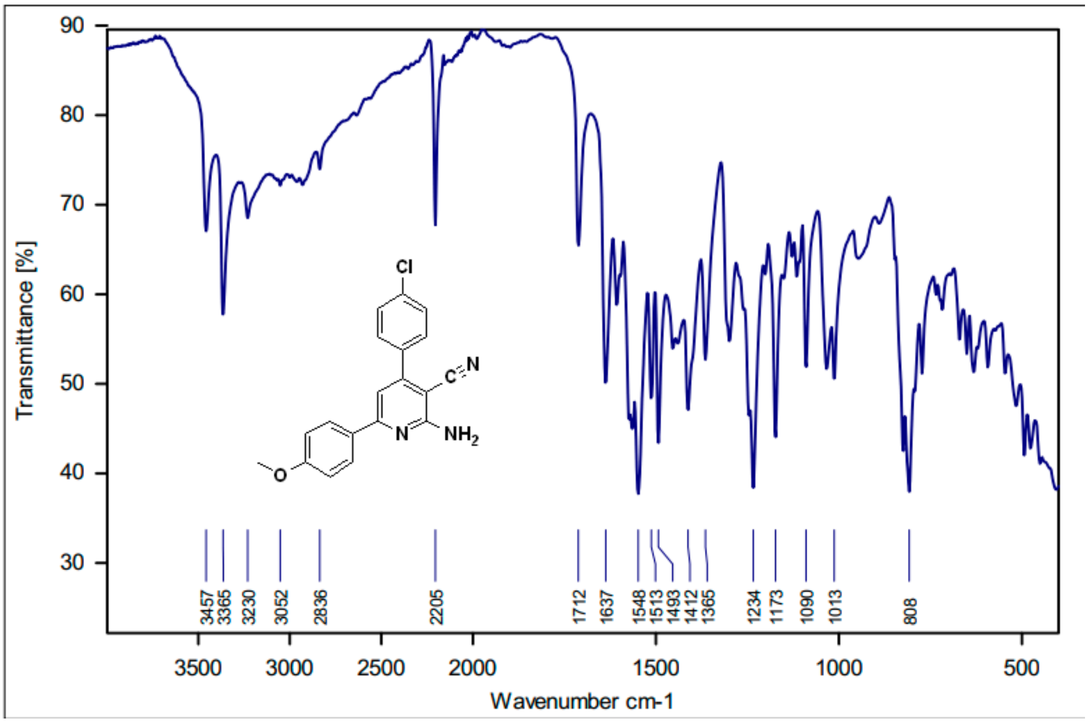


**Figure S4:** FTIR spectrum of compound **2**.

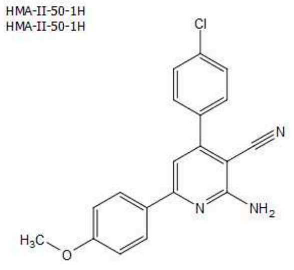


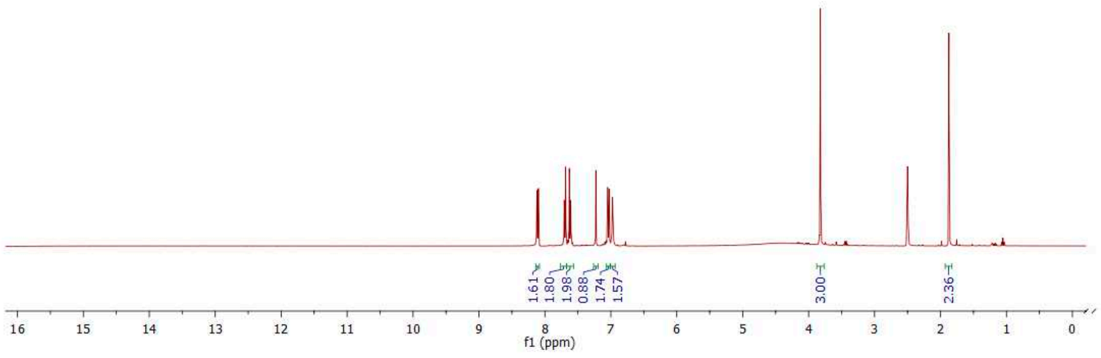


**Figure S5:** ¹H-NMR spectrum of compound **2**.

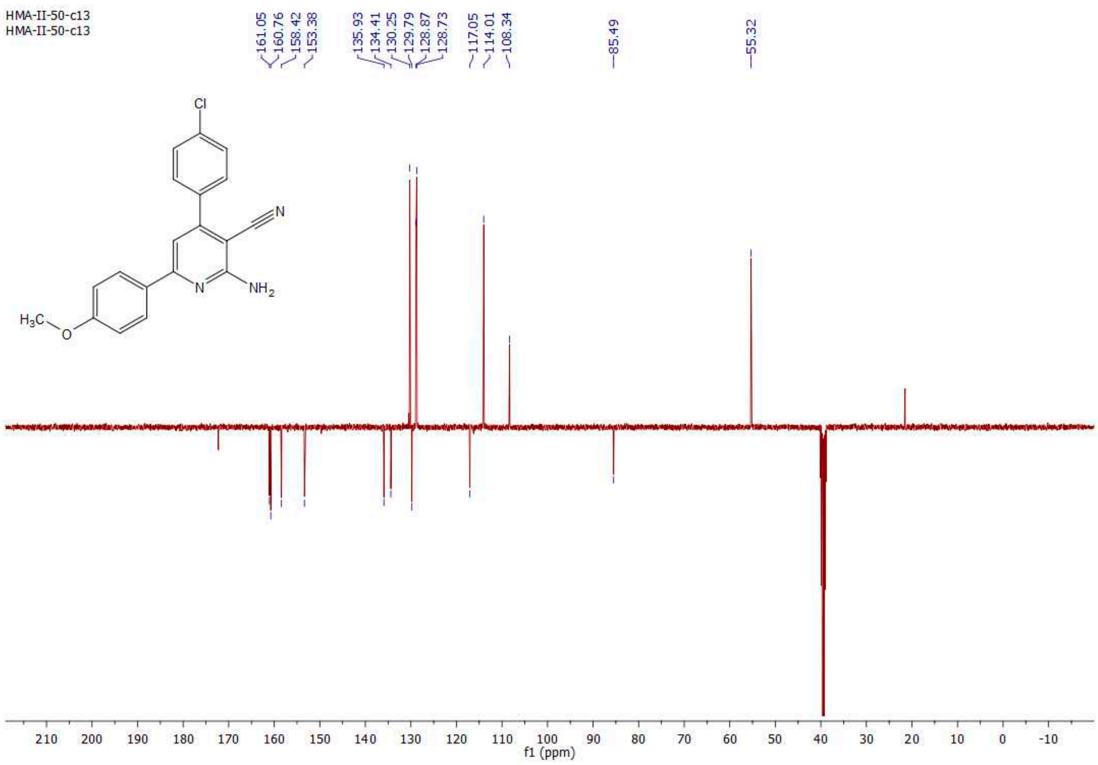


**Figure S6:** $^{13}$C-NMR spectrum of compound **2**.

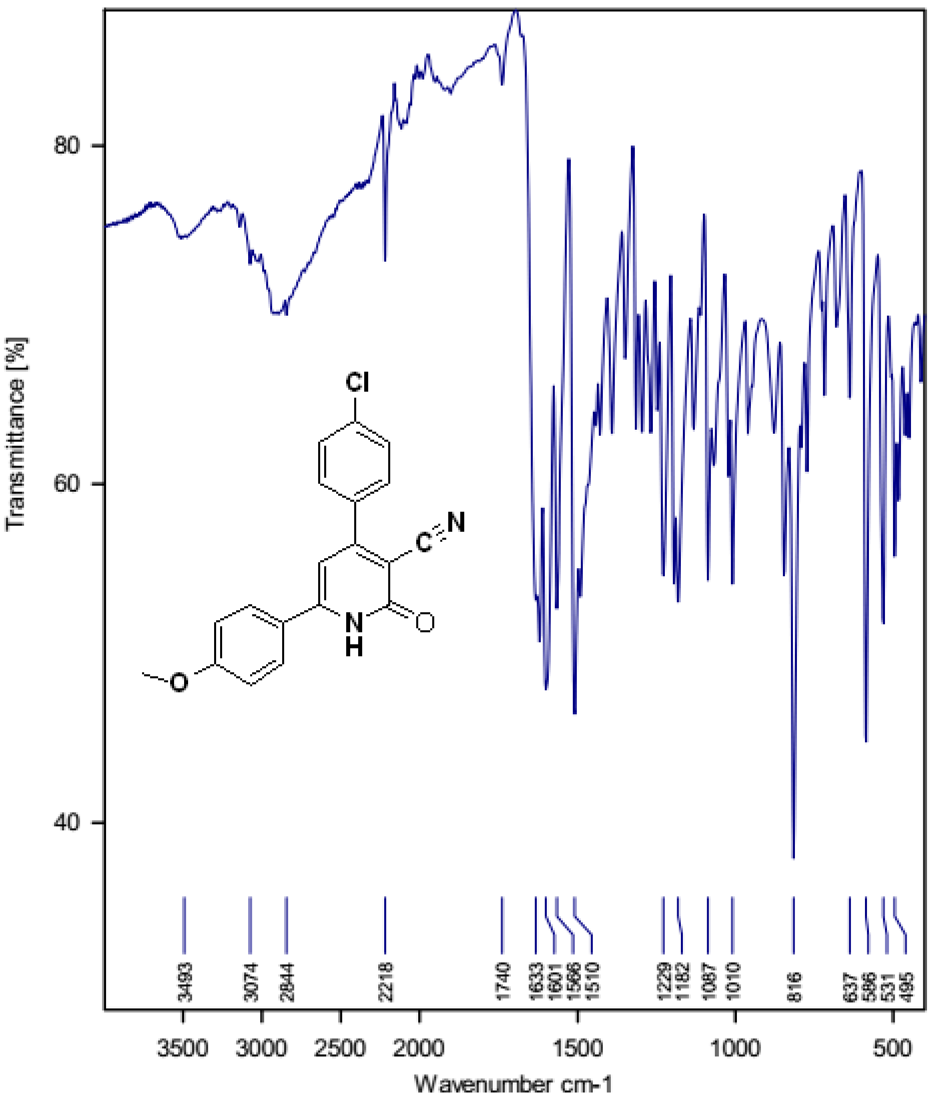


**Figure S7:** FTIR spectrum of compound **3**.

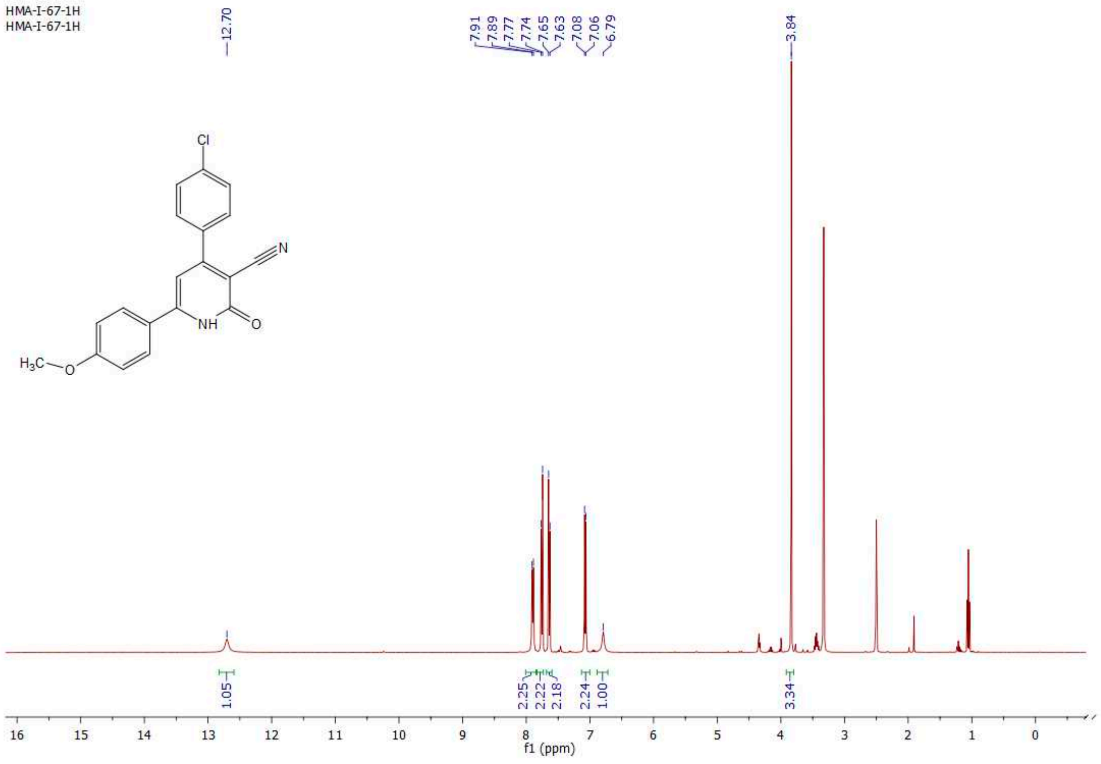


**Figure S8:** $^{1}$H-NMR spectrum of compound **3**.

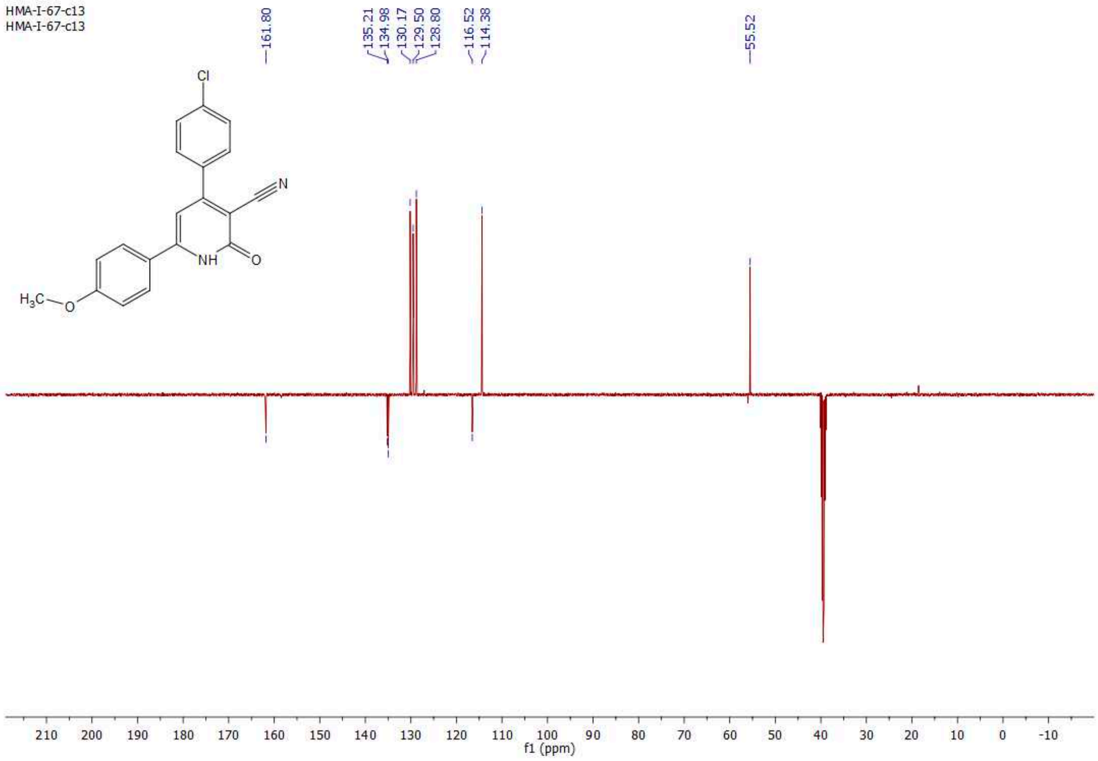


Figure S9: $^{13}$C-NMR spectrum of compound **3**.

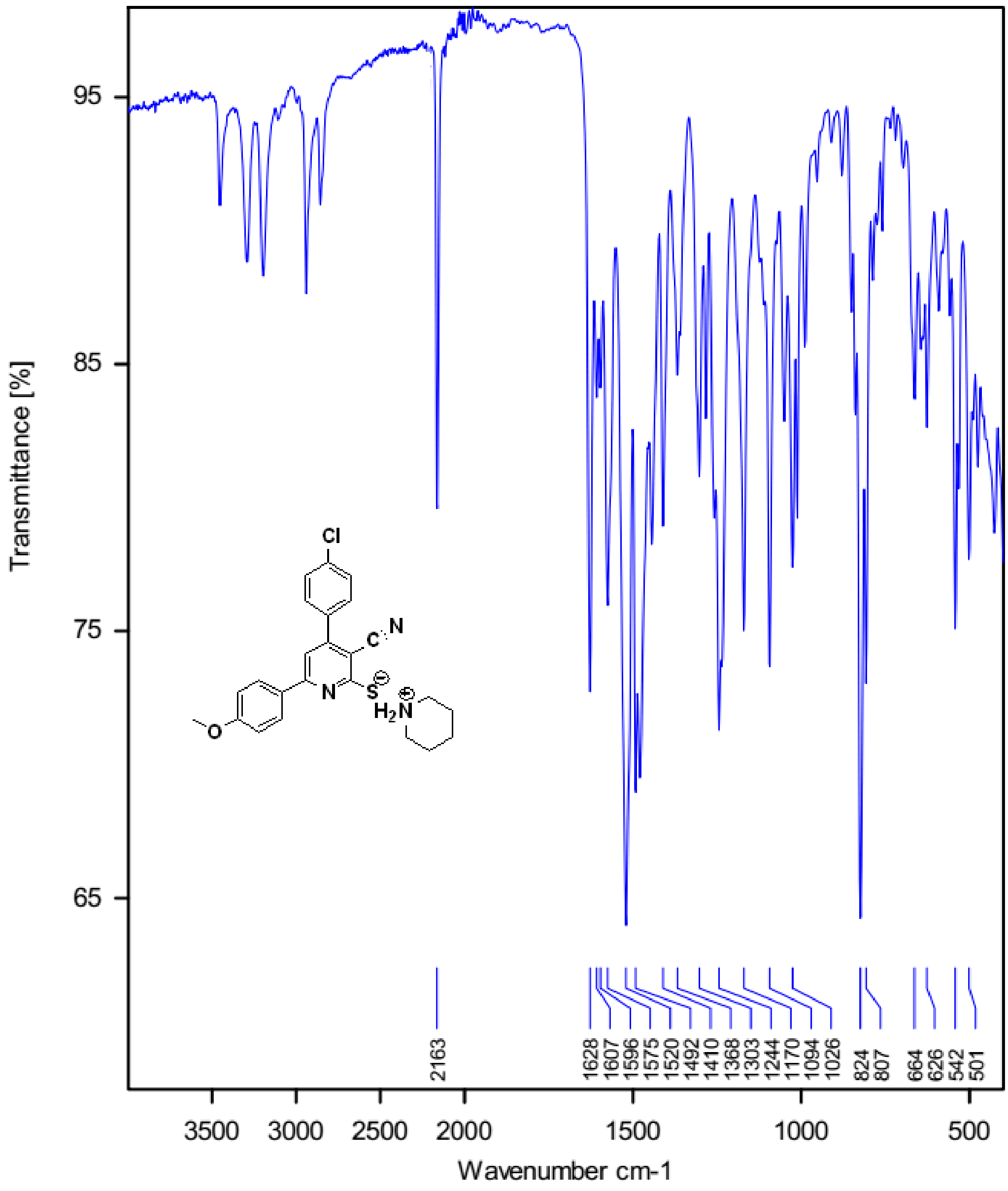


**Figure S10:** FTIR spectrum of compound **4a.**

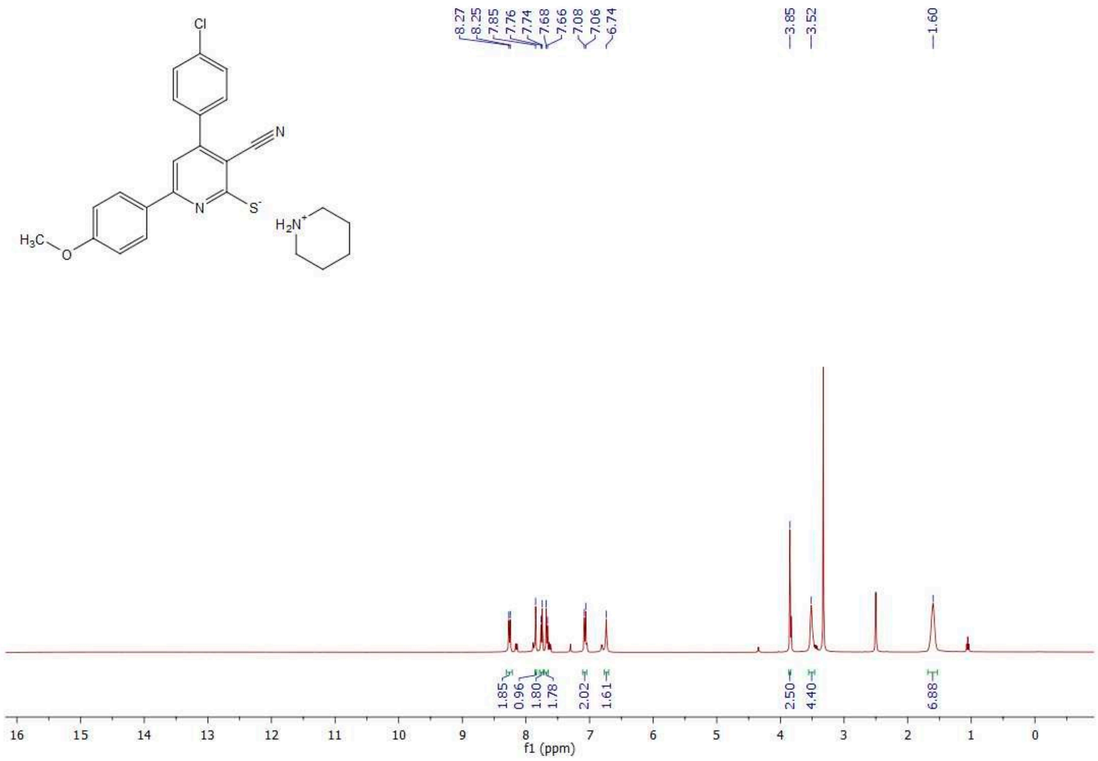

Figure S11: $^{1}$H-NMR spectrum of compound 4a.

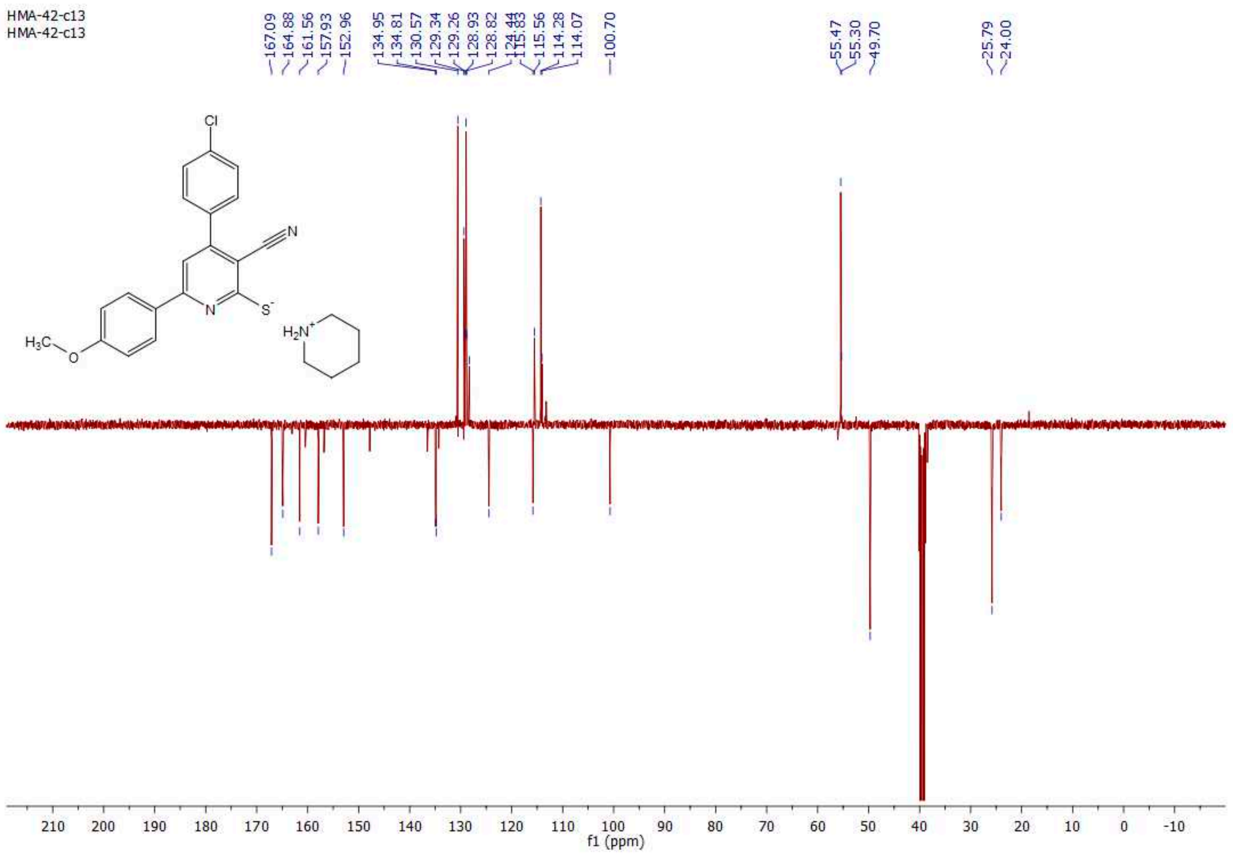


Figure S12: $^{13}$C-NMR spectrum of compound **4a**.

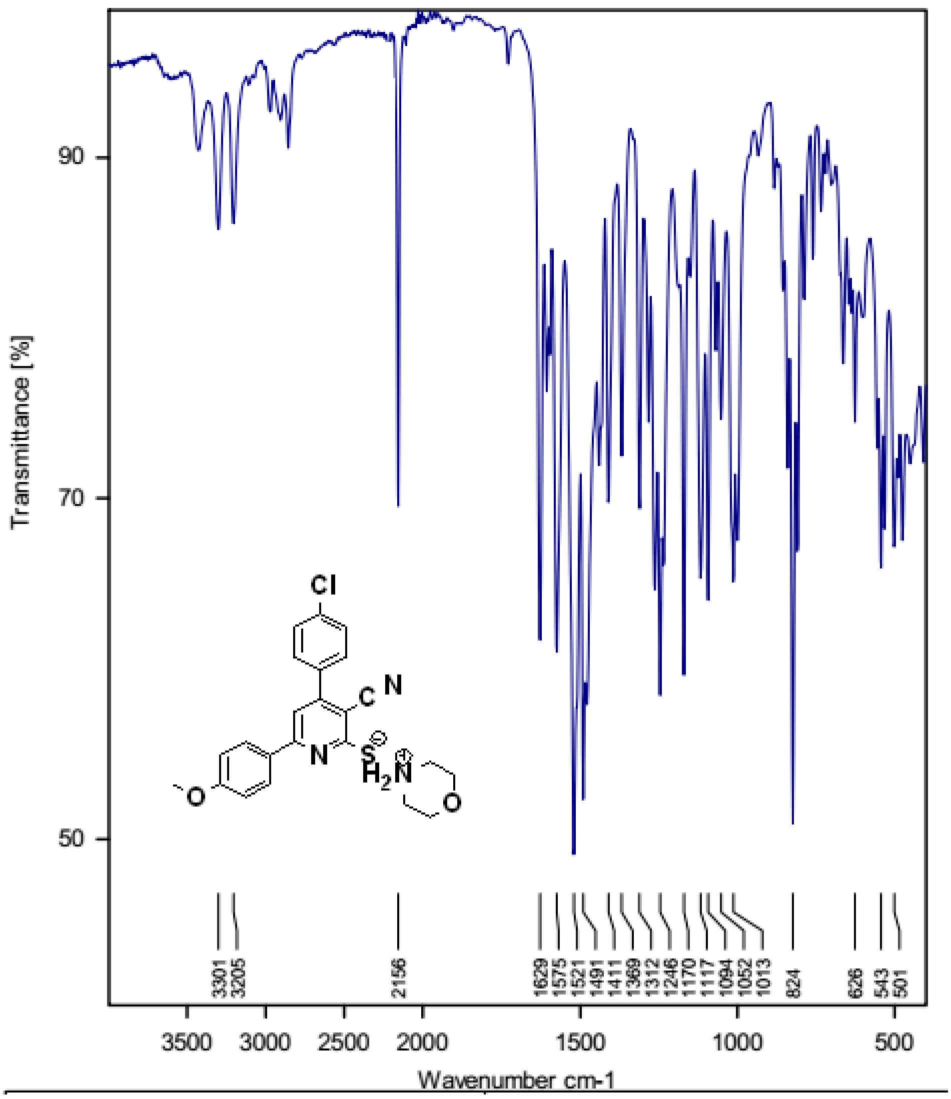


**Figure S13:** FTIR spectrum of compound **4b.**

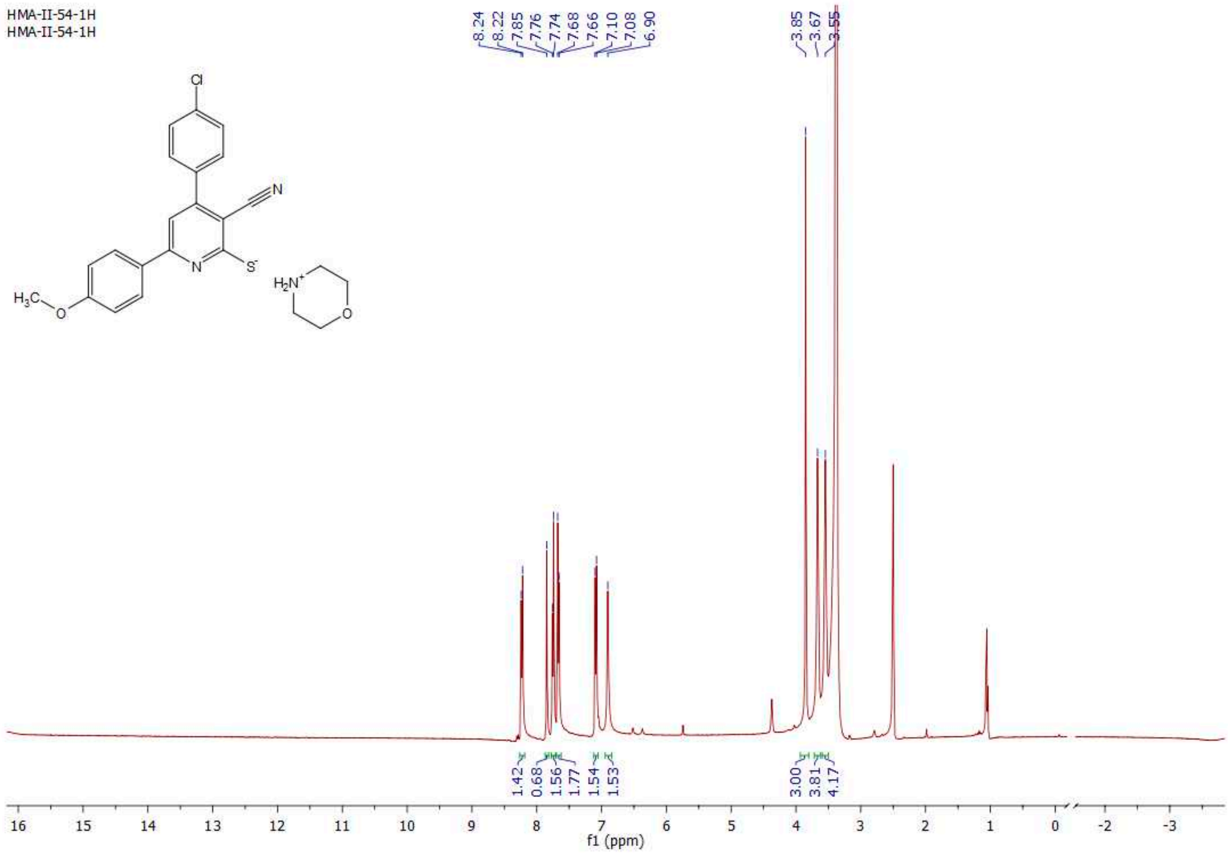


**Figure S14:** $^{1}$H-NMR spectrum of compound **4b.**

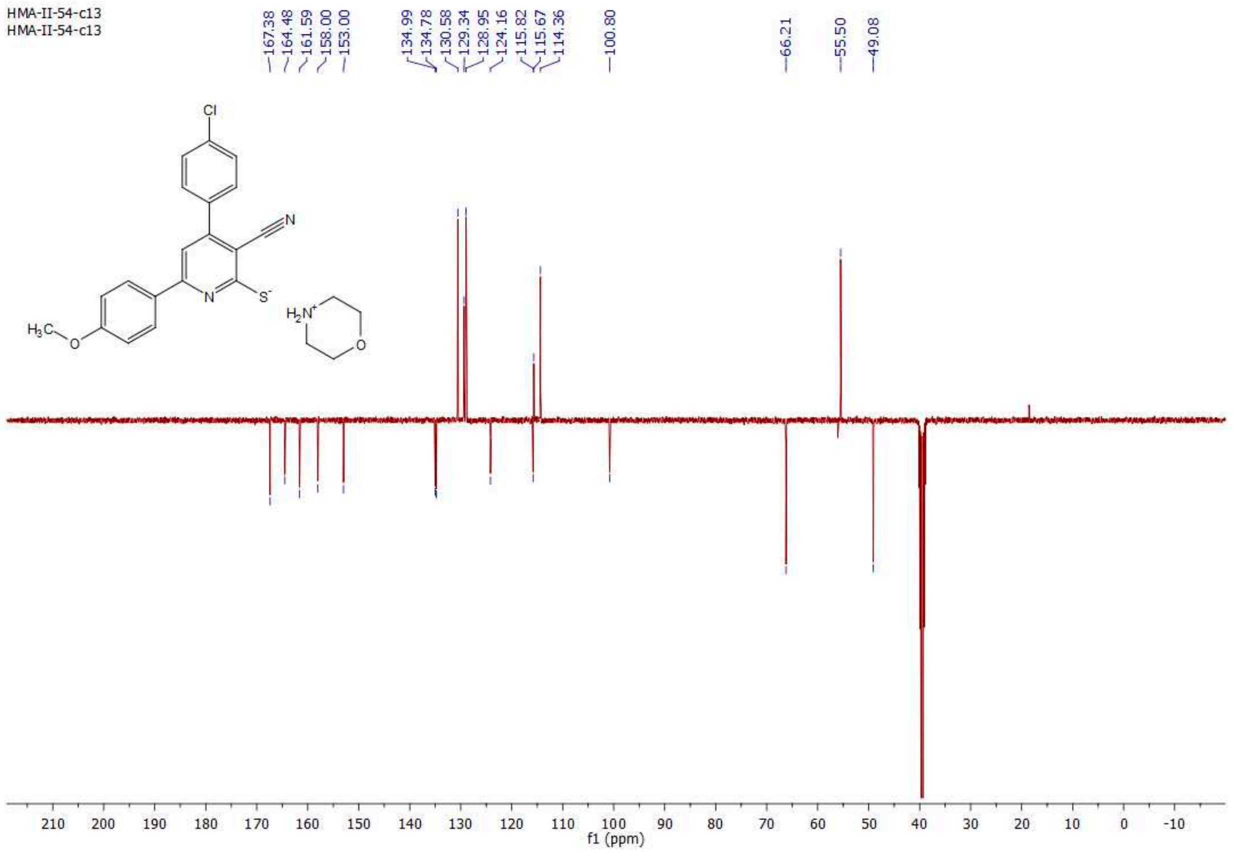


Figure S15: $^{13}$C-NMR spectrum of compound **4b**.

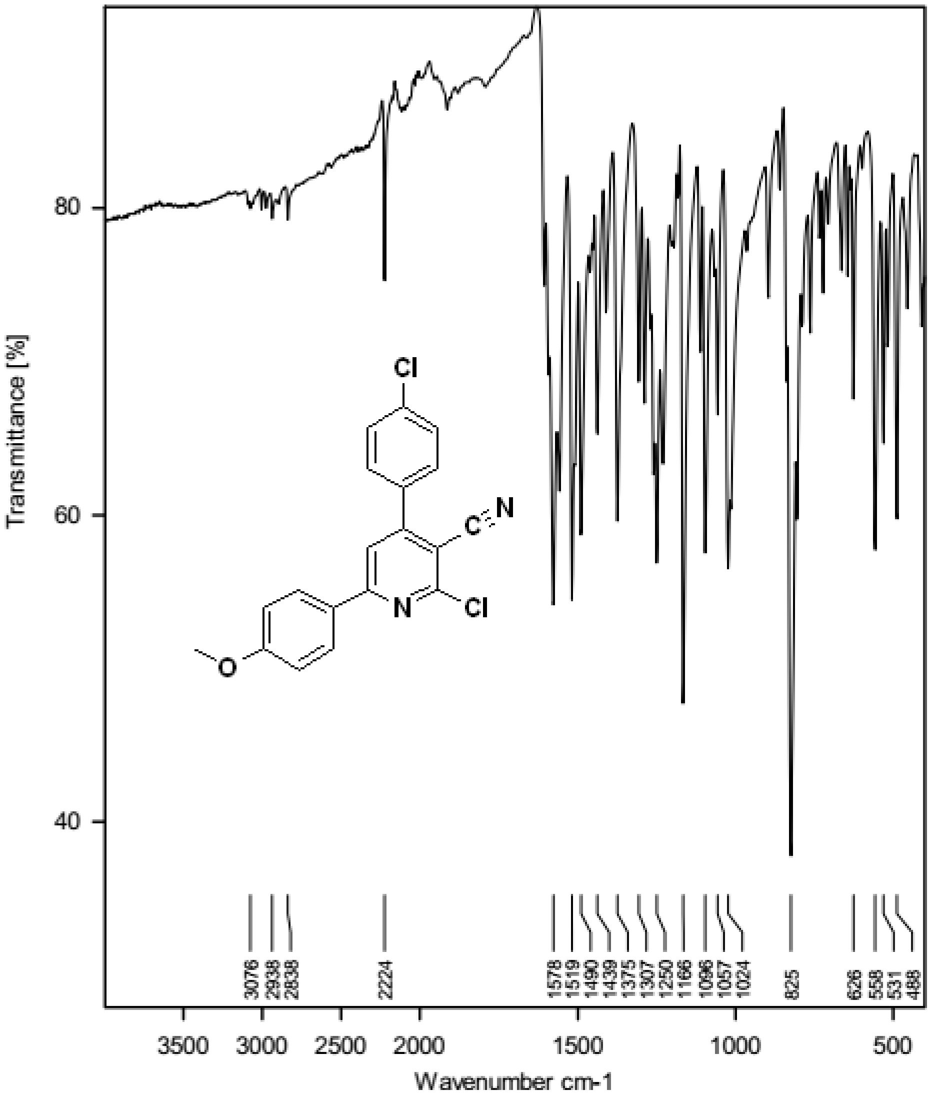


Figure S16: FTIR spectrum of compound 5.

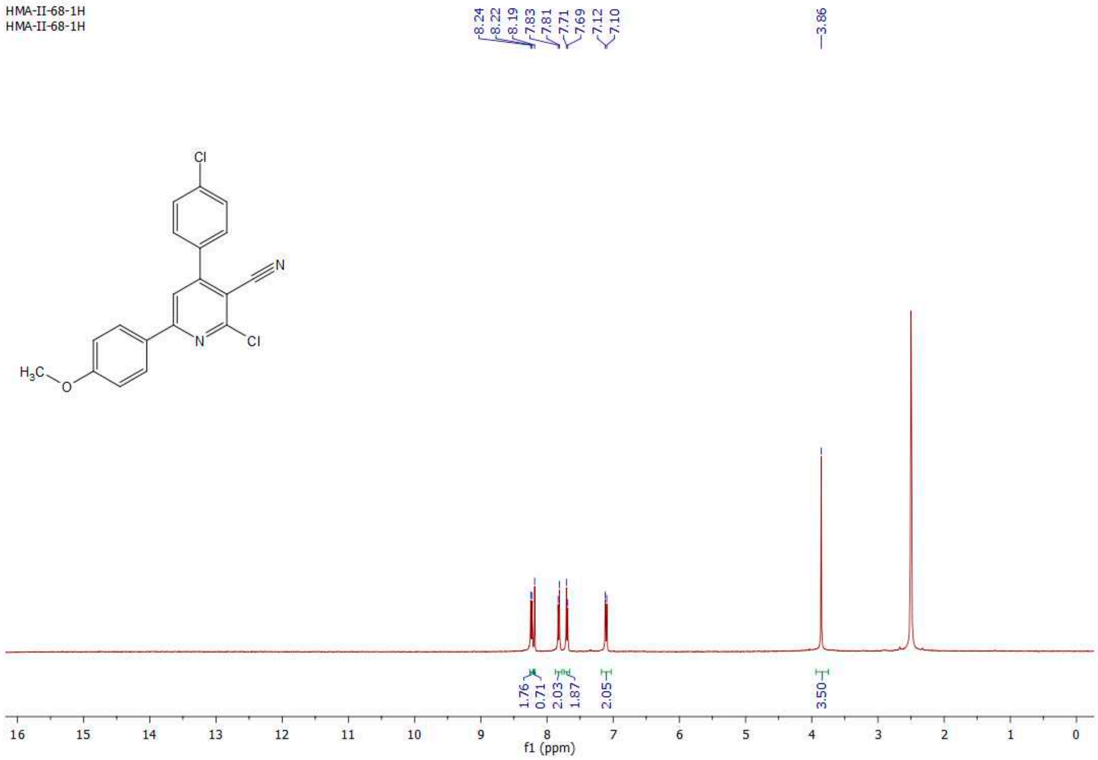


**Figure S17:** $^1$H-NMR spectrum of compound **5**.

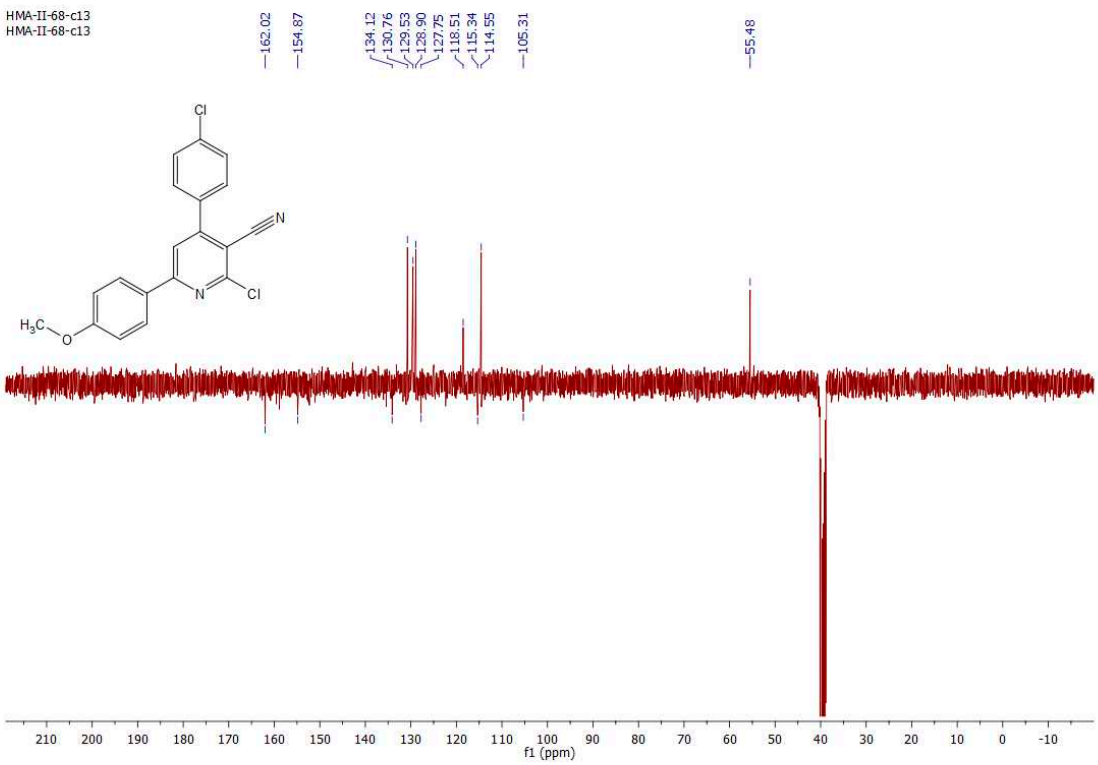


Figure S18: $^{13}$C-NMR spectrum of compound 5.

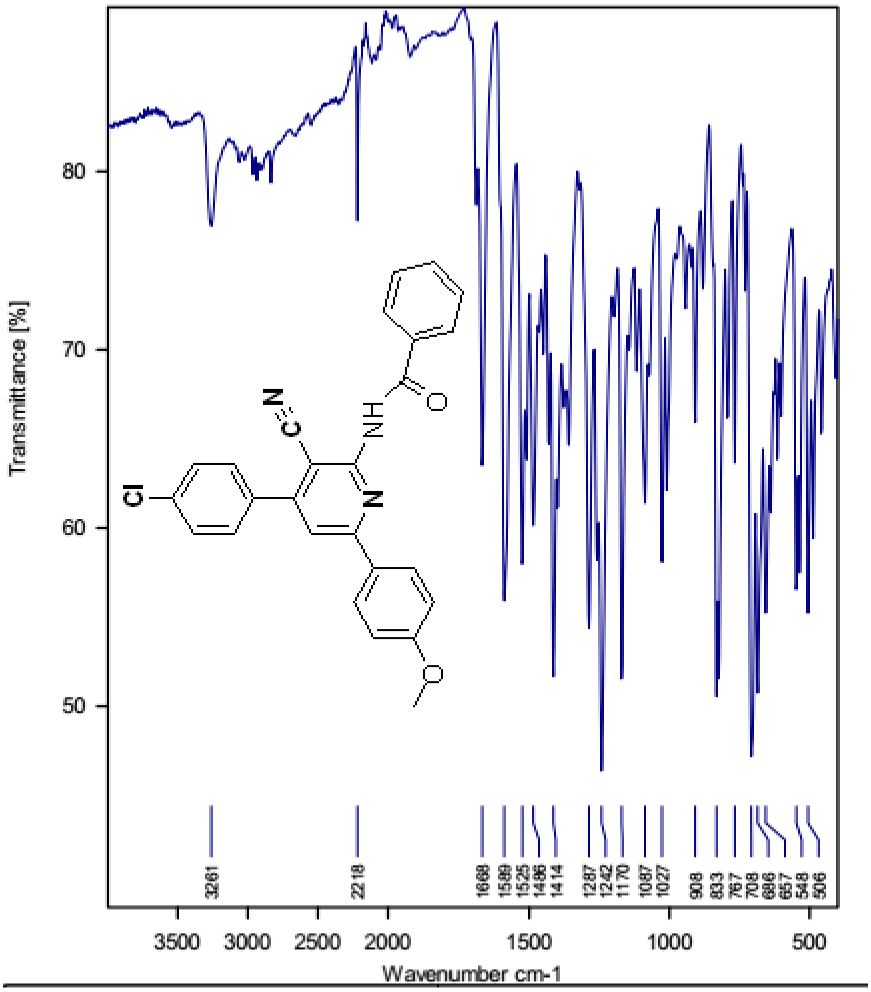


**Figure S19:** FTIR spectrum of compound **6a.**

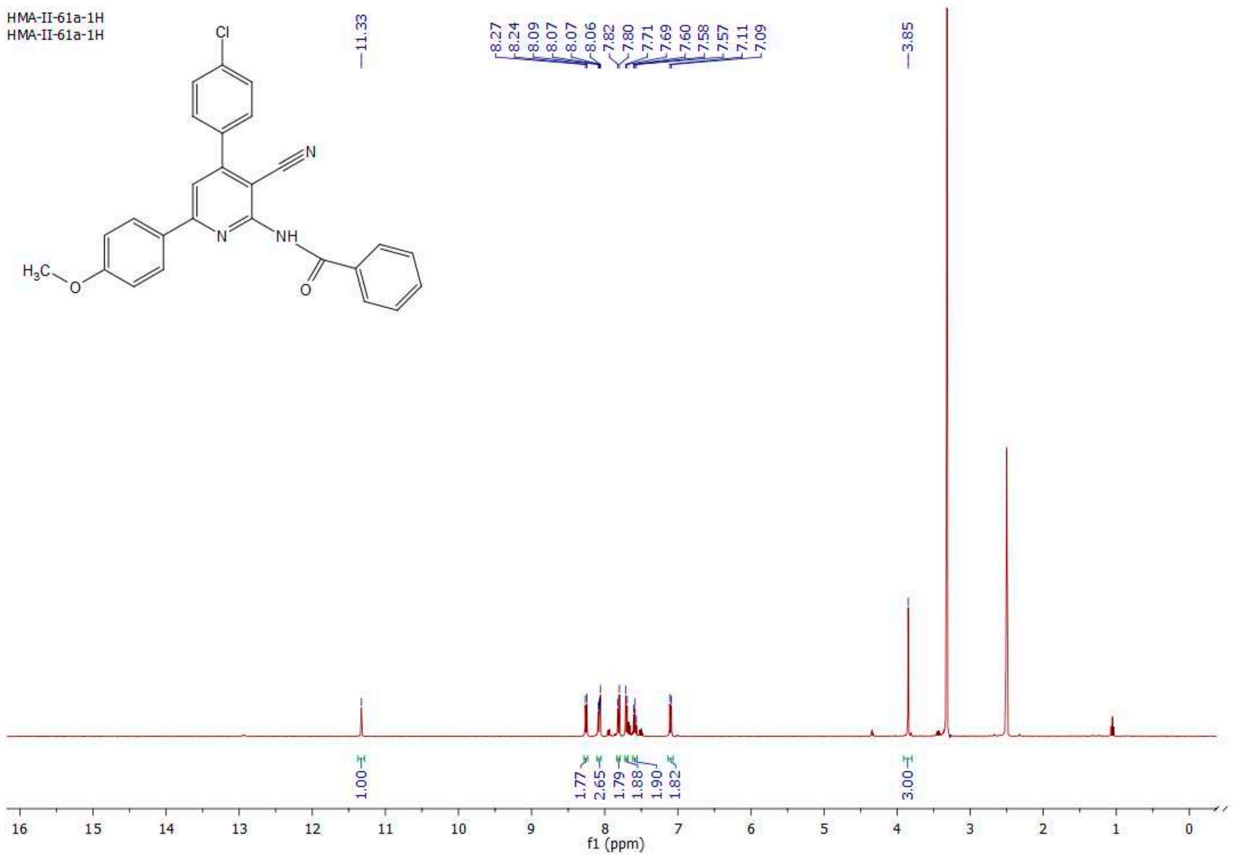


Figure S20: $^{1}$H-NMR spectrum of compound **6a**.

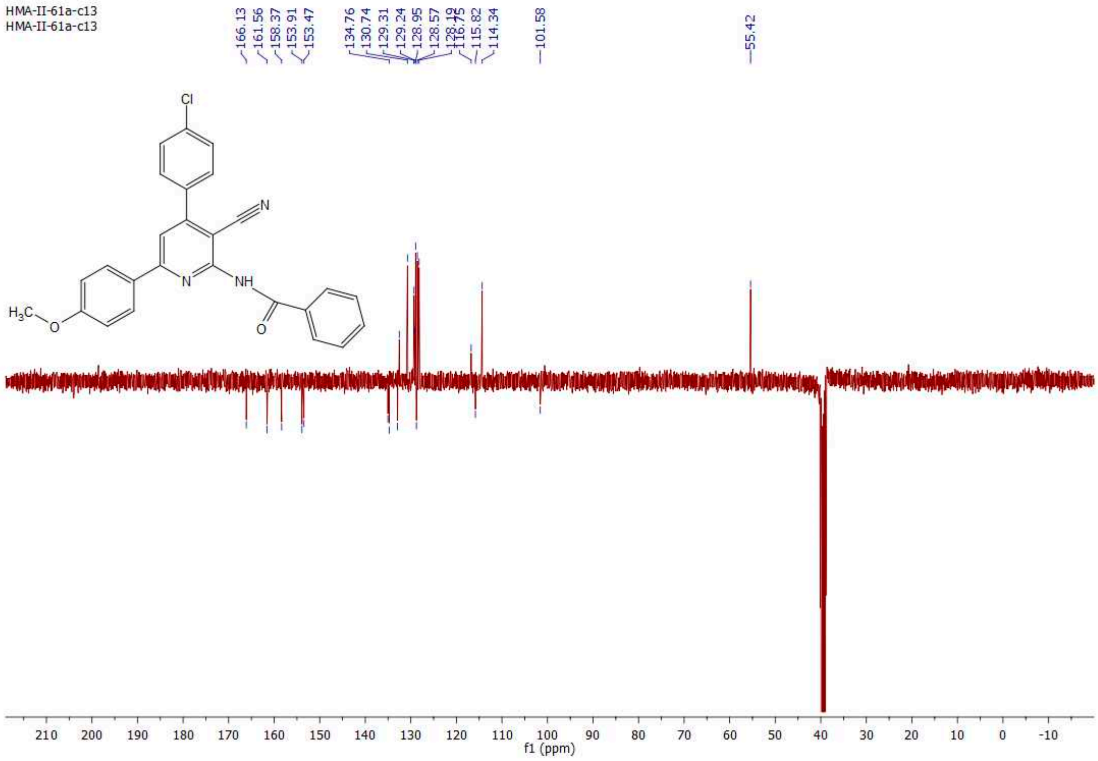


**Figure S21:** $^{13}$C-NMR spectrum of compound **6a**.

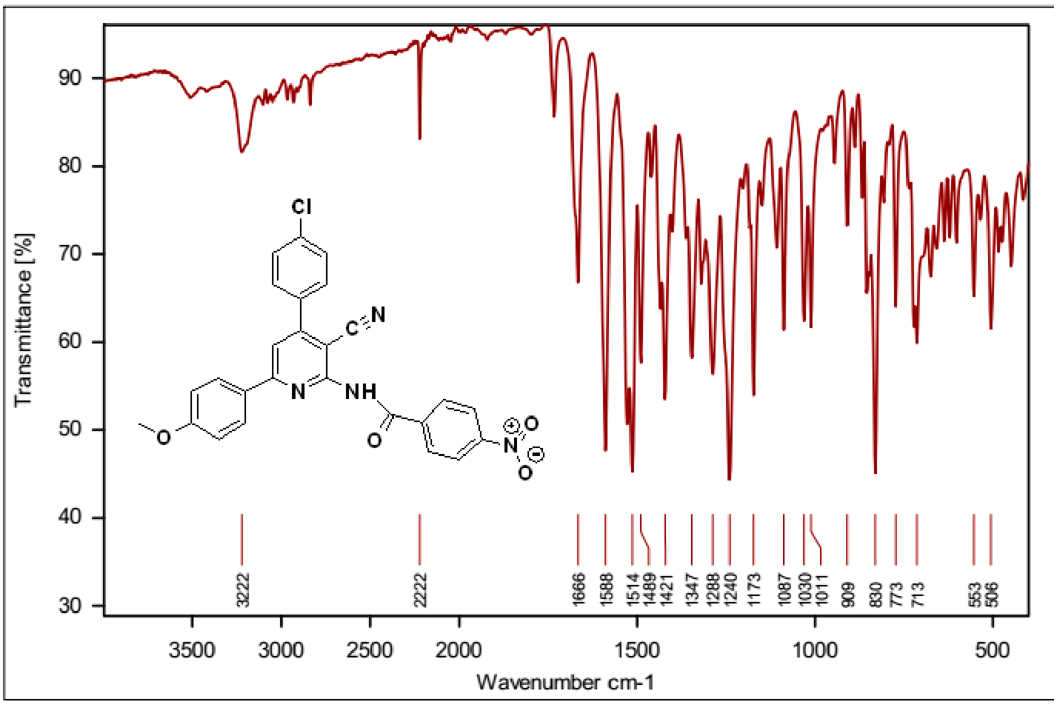


**Figure S22:** FTIR spectrum of compound **6b**.

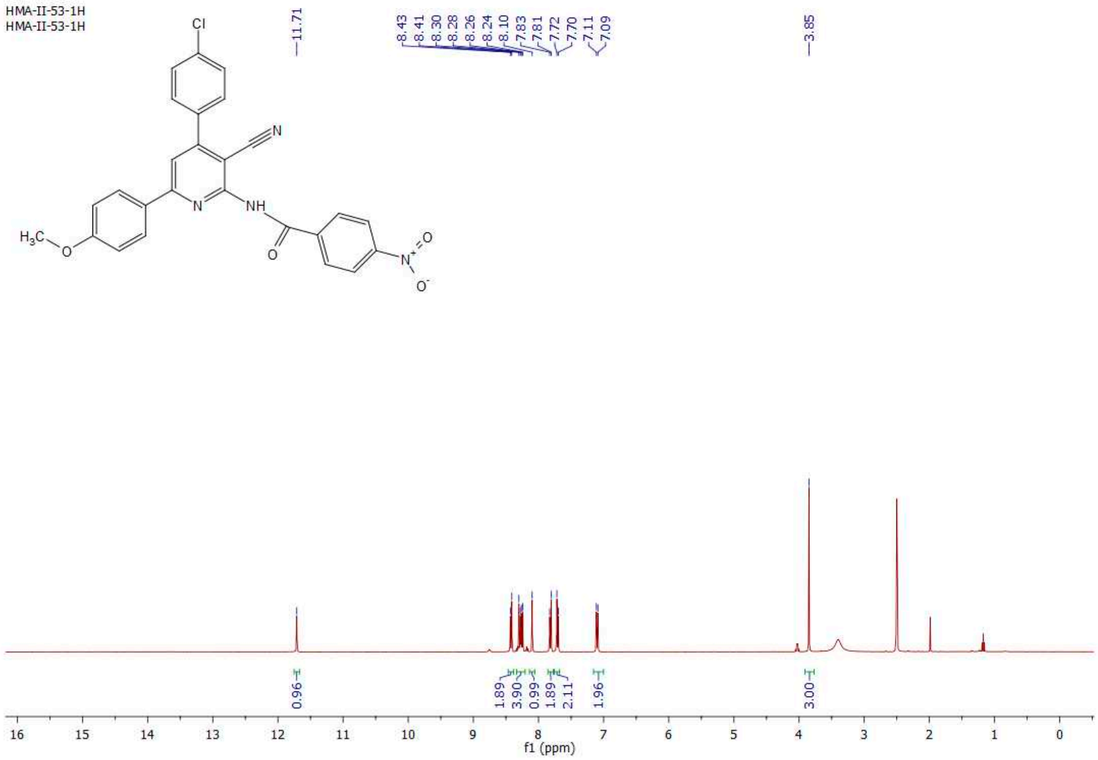


**Figure S23:** $^{1}$H-NMR spectrum of compound **6b**.

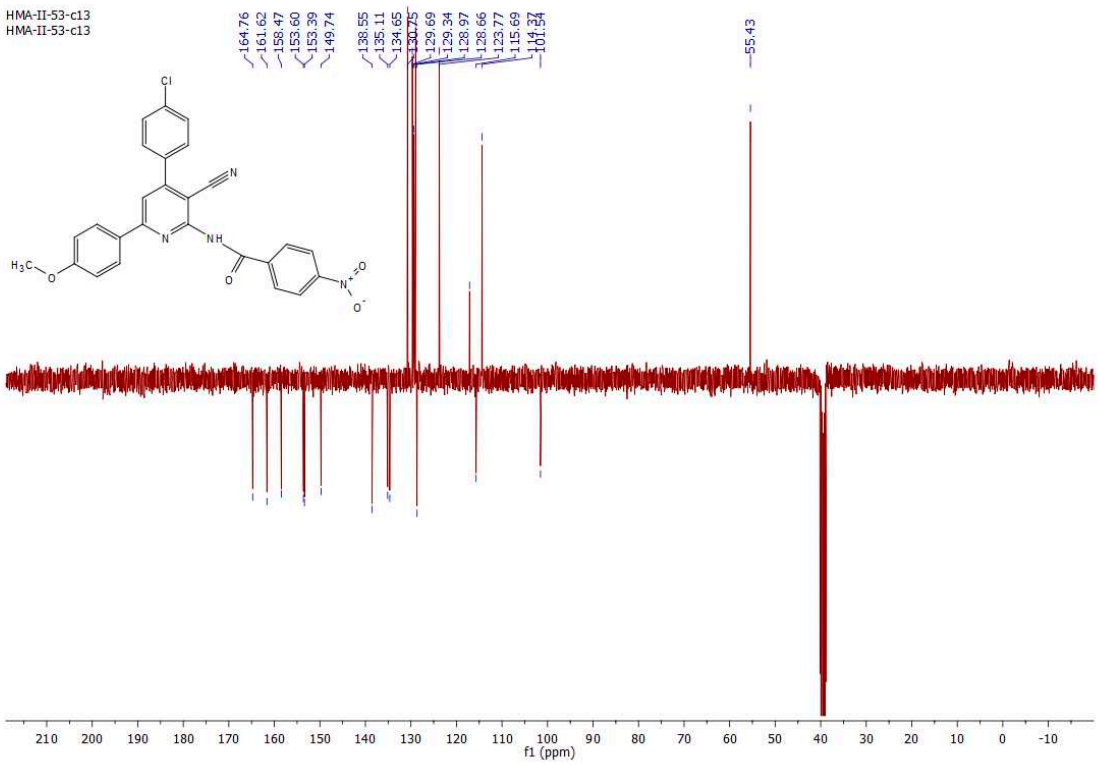


Figure S24: $^{13}$C-NMR spectrum of compound **6b**.

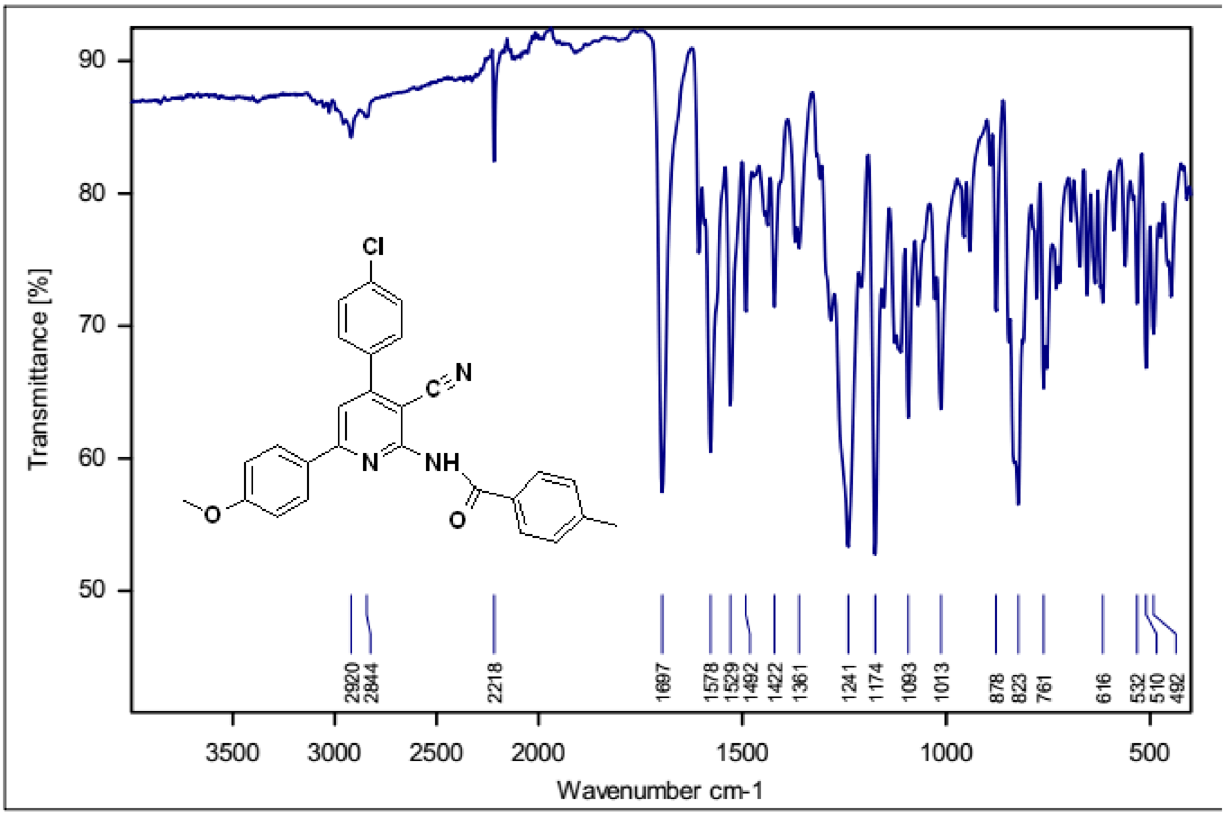


**Figure S25:** FTIR spectrum of compound **6c**.

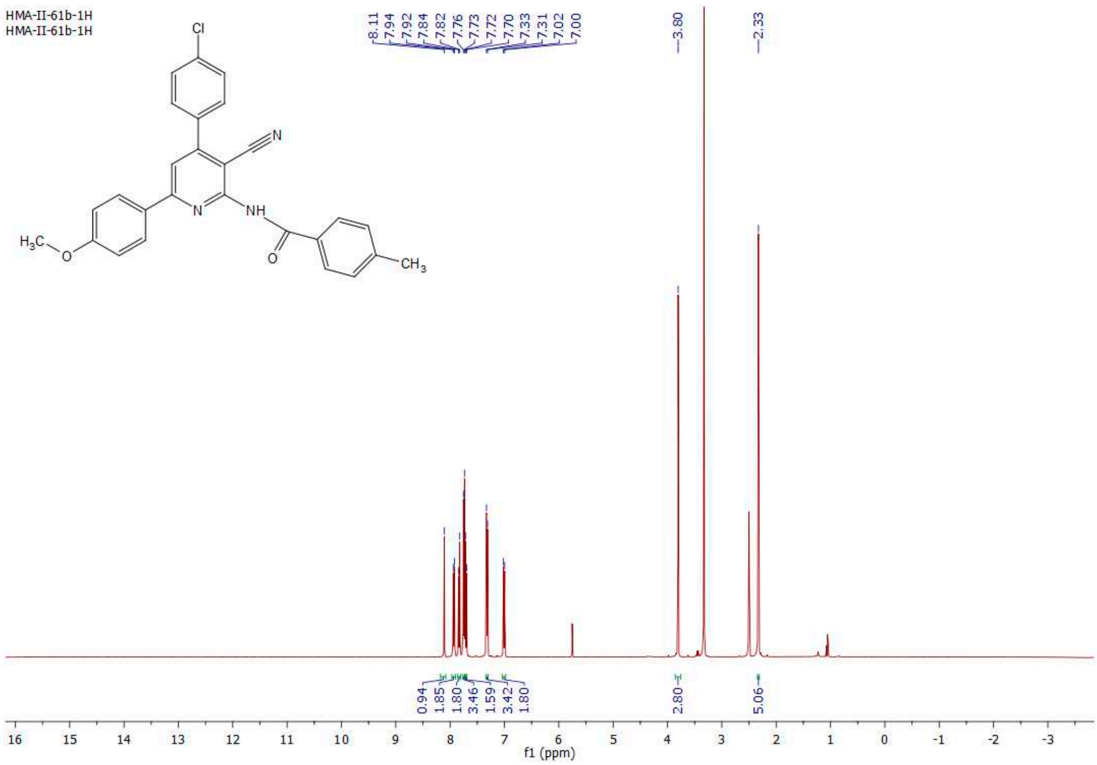


**Figure S26:** $^{1}$H-NMR spectrum of compound **6c**.

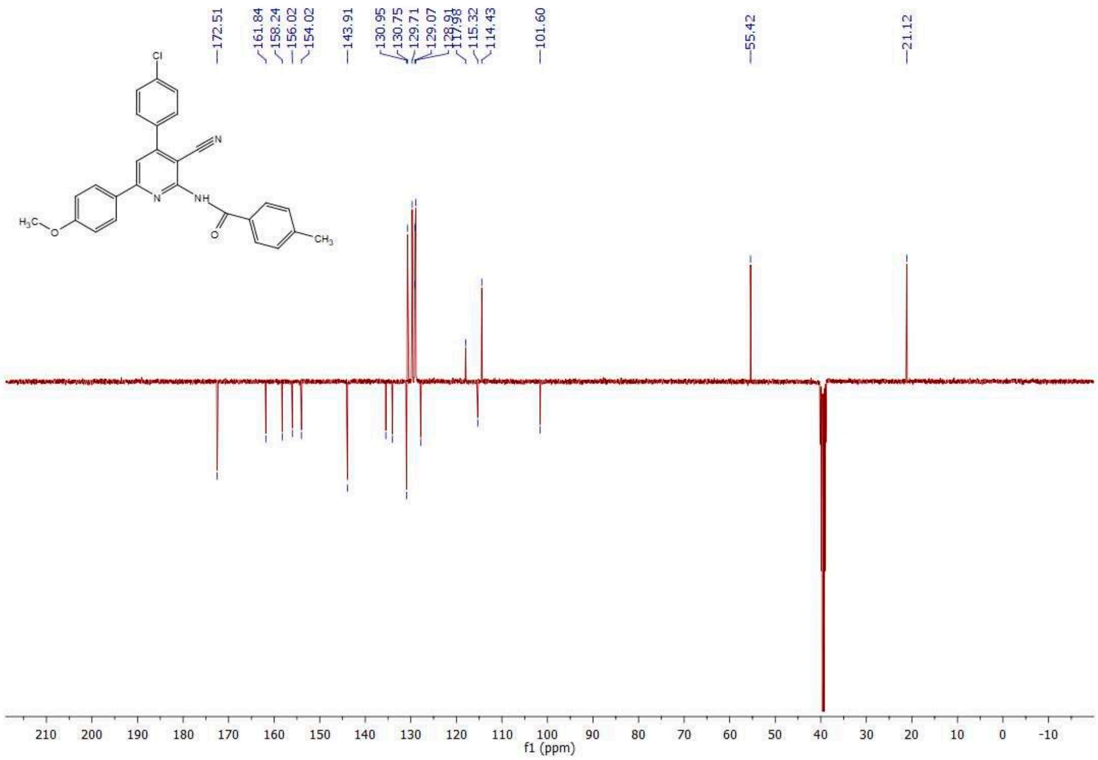


Figure S27: $^{13}$C-NMR spectrum of compound **6c**.

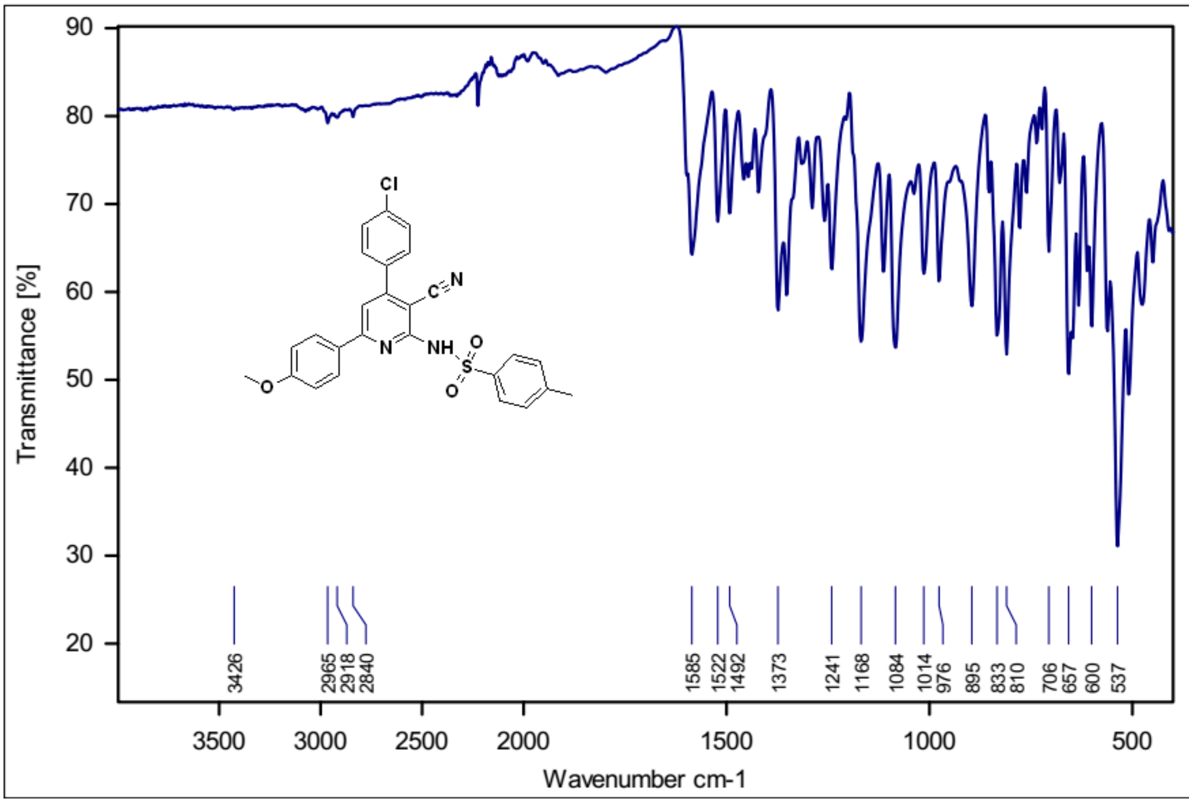


**Figure S28:** FTIR spectrum of compound **7**.

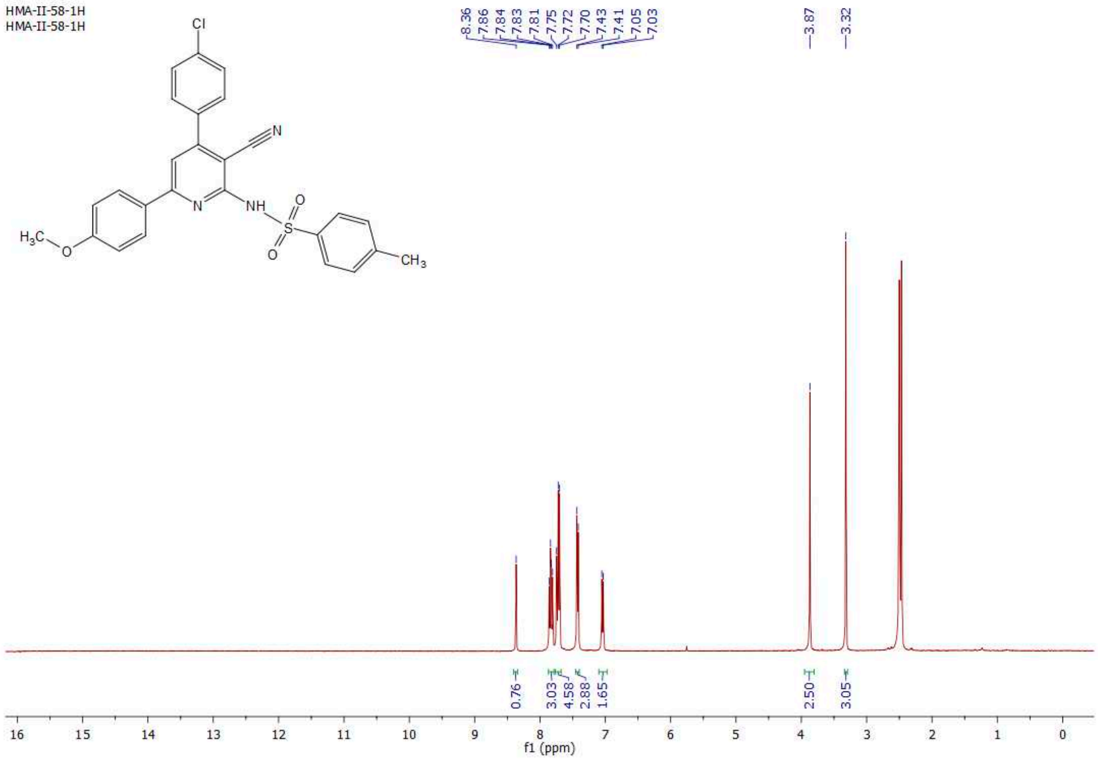


**Figure S29:** $^{1}$H-NMR spectrum of compound **7**.

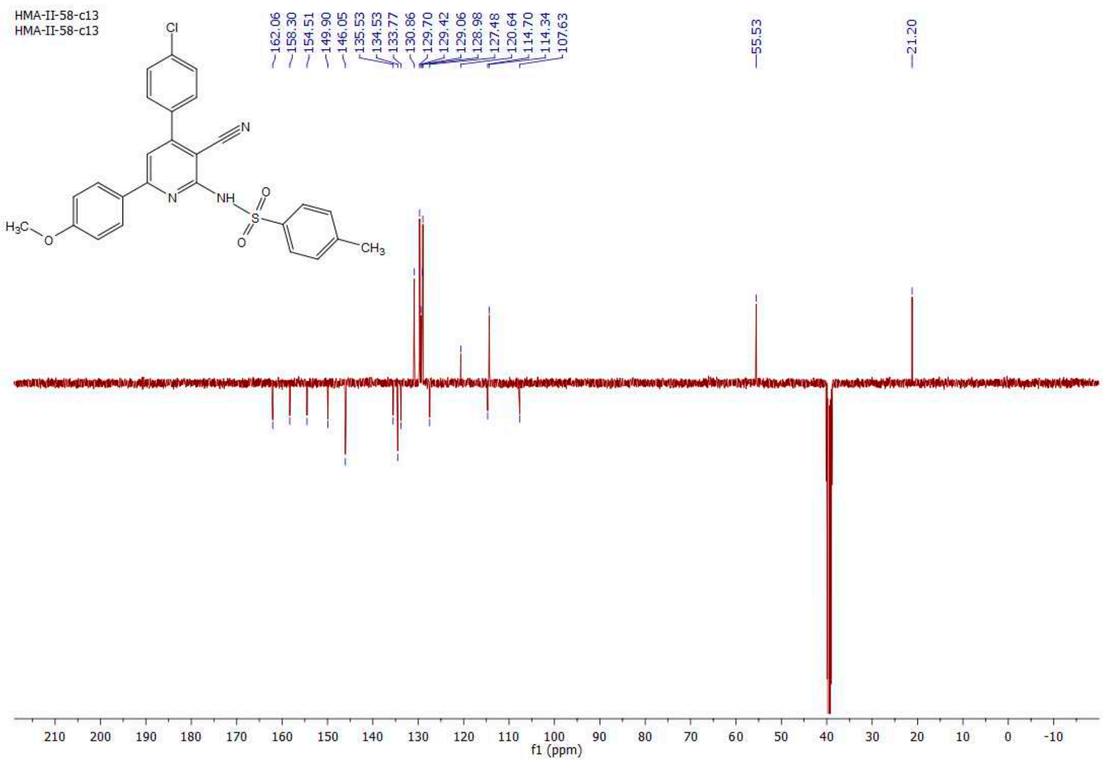


Figure S30: $^{13}$C-NMR spectrum of compound **7**.

RASAYELY

# EDITING CERTIFICATE

This document certifies that the below mentioned manuscript has been edited for English language by professional English-language Editors at RASAYELY. To verify this certificate, use the verification key below in the URL: https://rasayely.com/verify/

## Manuscript Title

Design, Synthesis, Characterization, and Molluscicidal Activity Screening of New Nicotinonitrile Derivatives against Land Snails, *M. cartusiana*

## Authors

Hend M. A. Maaroof, Albogami Bander, Reham A. I. Abou-Elkhair, Abdalla E. A. Hassan, Fatma I. Al-Akhrasy, Salem A. A. El-Massry, Eman Fayad, and Islam Zaki

## Date Issued

14 November 2022

## Certification Verification Key

WNHC-TWMY-VCT4-BAIH

http://rasayely.com

**Figure S31:** English certificate.

**Agricultural Research Center**

**Plant Protection Research Institute**

---

## Approval

This is certify that the protocol entitled:

**"Design, Synthesis, Characterization and Molluscicidal Activity Screening of New Nicotinonitrile derivatives against Land Snails,** ***M. cartusiana"***

Principle investigator. Dr. Hend Mohammed Ahmed Maaroof

Has been reviewed and approved by the animal car and use according to Plant Protection Research Institute, Agricultural Research Center, Dokki, Giza, Egypt.

First approval date: 12/ 2021

Expiration date: 12/ 2023

Please be advised that the animal care and use committee is limited to **two years**. Any technical or administrative changes to the approved protocol must be submitted in writing to the animal car and use committee for approval. Changes should not be initiated until written animal car and use committee approval is received. Any adverse events should be reported to the animal car and use committee as they occur. When you need to extend this protocol the renewal must be submitted for approval at least three months prior to the expiration date of this approval.

*Best Regards,*

Head of Plant Protection Research Institute

Sharkia branch

Prof. Dr. Ali Ahmed

## S4.1.1. Chemistry: General

Melting points were determined in open capillaries tube using Electrothermal Digital melting point apparatus and were uncorrected. The IR spectra were recorded on Shimadzu IR spectrophotometer, applied nucleic acid research center (ANARC), Faculty of Science, Zagazig University, Zagazig, Egypt. The $^{1}$H-NMR and $^{13}$C-APT NMR were performed on a Bruker 400 MHz (Bruker comp., MA, USA) at Applied Nucleic Acid Research Center (ANARC), Faculty of Science, Zagazig University, Egypt. Elemental analyses were performed on Elementar, Vario El, Microanalytical unit, Cairo, Egypt and were found within ±0.4% of the theoretical values.

## S4.2. Biological studies

### S4.2.2. Biochemical assays

#### S4.2.2.1. Preparation of samples for biochemical assay

Samples used for biochemical assays were collected at 3 days post treatment of $LC_{50}$ for **2, 4a, 4b**, and Acetamiprid compounds. Untreated snails were used as control. Samples were homogenized in distilled water using a teflon homogenizer. The homogenates were centrifuged at 5000 rpm for 10 minutes at 5 ºC, the supernatants were immediately assayed to determine the total soluble protein, the activities of aspartate aminotransferase (AST), alanine aminotransferase (ALT), and acetylcholinesterase (AChE).
